# Multimodal graph neural network with large language models for node and link prediction

**DOI:** 10.3389/frai.2026.1758852

**Published:** 2026-06-19

**Authors:** Bo Peng, Huan Xu, Xiangjiu Che

**Affiliations:** College of Computer Science and Technology, Jilin University, Changchun, Jilin, China

**Keywords:** graph editor, graph neural network, large language models, multimodal, oversmoothing

## Abstract

**Introduction:**

Graph data representation is widely applicable in numerous real-world scenarios, and recent advances in graph neural networks (GNNs) have enabled effective modeling of complex associations in graph-structured data. However, GNNs are often constrained by the over-smoothing problem, which reduces their ability to distinguish node representations. In contrast, large language models (LLMs) are effective at capturing semantic contexts from textual data but are inherently limited in encoding structural information from graphs.

**Methods:**

We propose multimodal and semantic-enhanced GNN (M2GNN), an LLM-enhanced graph learning framework for node classification and link prediction on graphs with structural and textual feature modalities. M2GNN is not designed as a general-purpose graph multimodal large language model. Instead, it focuses on a controlled graph-text prediction setting, in which LLM-derived semantic embeddings guide candidate edge refinement, stable positional encodings are applied to the refined graph, and graph-based and language-informed predictors are adaptively fused.

**Results:**

Empirical results and ablation analyses show that M2GNN achieves competitive or improved performance on the evaluated benchmarks. The analyses further clarify the contribution of semantic edge refinement, stable structural encoding, and graph-language prediction fusion to node and link prediction performance.

**Discussion:**

Rather than claiming novelty for each individual component, this work demonstrates how semantic edge refinement, stable structural encoding, and adaptive graph-language fusion can be integrated into a practical pipeline for graph-text prediction tasks. The findings suggest that combining LLM-derived semantic information with structure-aware graph learning can improve prediction robustness while maintaining a clearly defined task-specific scope.

## 1 Introduction

Graphs offer a highly flexible and powerful means of representing data, naturally depicting intricate entity relationships and network structures (Ji et al., 2021), and are widely used across various domains, including social network analysis, knowledge graphs, and natural language processing (NLP) (Zhang et al., 2020). In deep learning, graphs can be used as input data for numerous graph-structured data sets (Zhang et al., 2020). Among these, graph neural networks (GNNs) have demonstrated robust capability in processing graph-structured data, offering advantages in modeling complex relationships, flexibility, and semi-supervised learning (Zhou et al., 2020). GNNs can efficiently handle complex relationships and structural information in graph data (Gilmer et al., 2020). GNNs operate through a message-passing mechanism, enabling each node to reflect information from its neighbors and the broader graph (Gilmer et al., 2020). Concurrently, advances in computational power and data resources have driven expansion in the scale of large language models (LLMs) (Chavan et al., 2024). LLMs are proficient in various NLP tasks, including text generation, translation, summarisation, question-answering, and dialogue systems (Naveed et al., 2023). These neural network-based models have demonstrated remarkable multilingual capabilities owing to extensive pre-training and fine-tuning on diverse tasks (Naveed et al., 2023; Kumar, 2024). They utilize the Transformer architecture, which facilitates more efficient text processing and generation through attention mechanisms (Kumar, 2024). Their ability to process and generate text through training on extensive, multilingual corpora makes them suited for tasks requiring nuanced language comprehension, context retention, and linguistic creativity (Kumar, 2024).

Nevertheless, LLMs and GNNs face limitations. Key challenges relate to computational resource requirements, model bias, factual accuracy, and interpretability (Tong et al., 2024). LLM-generated text occasionally contains factual inaccuracies or misleading information—a significant concern in domains where precise information is critical, such as medicine and law (Laban et al., 2023). Ensuring the accuracy and reliability of generated content remains a significant challenge (Wang et al., 2024). Secondly, LLMs perform sub-optimally when confronted with data encompassing intricate relationships and dependency structures (Hu et al., 2024; Agrawal et al., 2025). This limitation stems from their optimization for sequential text, making them less effective at capturing the intricacies of complex graph structure information (Hu et al., 2024; Agrawal et al., 2025). Conversely, GNNs may be susceptible to oversmoothing when processing deep structures (Li et al., 2018). This phenomenon occurs when distinct node representations converge toward a uniform state after traversing multiple layers of message passing (Rong et al., 2020). This convergence diminishes the model's ability to discern subtle differences, reducing its discriminative power (Rong et al., 2020; Nt and Maehara, 2019; Rusch et al., 2023). This challenge is prevalent in deep GNN models, which exhibit limited depth and expressiveness (Rusch et al., 2023).

**Figure 1:**
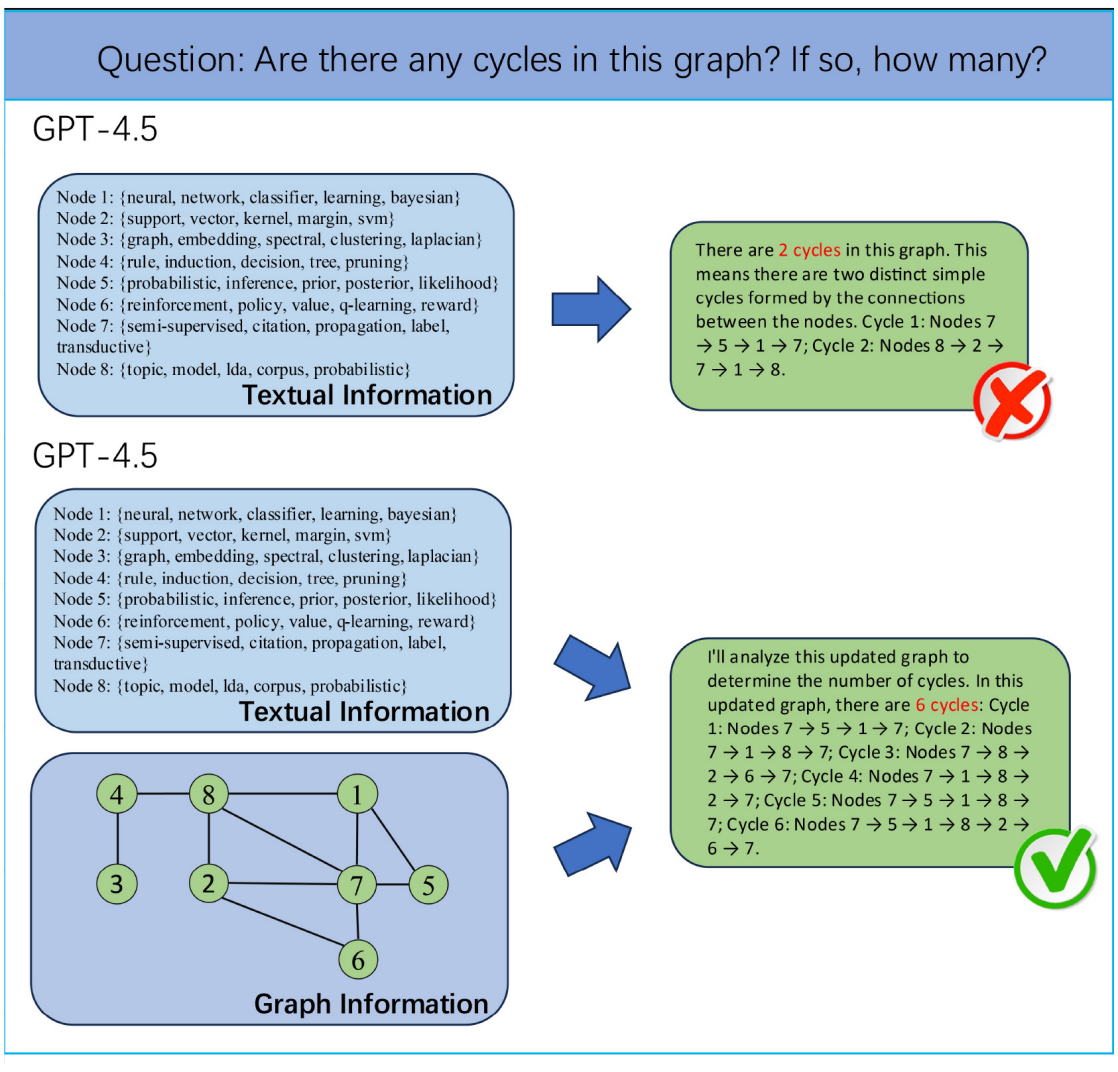
The impact of structural information on cycle detection in graphs. **(Top)** Text-only inputs follow the same data fields as our datasets and lead to unreliable judgments; **(Bottom)** adding the explicit graph enables correct cycle counting.

Conversely, we posit that a multimodal approach can integrate data from GNNs and LLMs. Multimodal approaches enable the integration of information from disparate modalities, offering a more comprehensive and multidimensional representation of the data (Xu et al., 2024). This fusion can enhance model robustness and generalization capabilities (Xu et al., 2024). Figure 1 illustrates the role of structural information in cycle detection in graphs, comparing model performance when relying solely on textual descriptions vs. when incorporating graphical representations. The results highlight that while GPT-4.5 can provide reasonable inferences based on text alone, its accuracy improves significantly with explicit graph structures. Multimodal approaches leverage complementary information inherent to different modalities, facilitating enhanced contextual comprehension and semantic reasoning (He et al., 2024). Nevertheless, integrating multimodal data necessitates the development of sophisticated model architectures and fusion strategies (He et al., 2024). Furthermore, extracting and fusing features from different modalities to avoid information loss and redundancy is challenging (Zhang et al., 2024). Data from different modalities often vary in noise levels and quality, necessitating mechanisms to manage inconsistencies and ensure the integrity of the fused data set (He et al., 2024; Gao et al., 2020). Another challenge is the high cost of acquiring and labeling multimodal data, particularly when a substantial quantity of high-quality data is needed (Xu et al., 2024; He et al., 2024).

To address these challenges, we propose multimodal and semantic-enhanced GNN (M2GNN), an LLM-enhanced graph learning framework for node classification and link prediction. In this work, the term “multimodal” refers primarily to the integration of graph-structural information and textual or feature-based semantic information. M2GNN targets static graph prediction tasks where semantic information from LLMs can complement graph topology under a controlled computational budget. M2GNN combines three components in a single prediction pipeline: LLM-guided semantic edge refinement, stable positional encoding on the refined graph, and adaptive fusion of graph-based and language-informed predictors. We do not claim that these components are individually new. The main contribution is their task-specific integration for graph-text node classification and link prediction, together with empirical analysis of when each component contributes to performance.

This work makes three main contributions:

(1) A graph-text prediction framework. We propose M2GNN for node classification and link prediction on graphs with structural and textual feature modalities. The framework uses LLM-derived semantic information for controlled edge refinement and combines it with structure-aware graph prediction.

(2) Adaptive fusion of graph and language predictors. M2GNN jointly trains a structure-aware GNN predictor and a language-informed LLM predictor and fuses them in a shared prediction space via adaptive weighting, leveraging complementary inductive biases for robust predictions under noisy and incomplete graph modalities.

(3) Component-level evaluation and updated positioning. We evaluate M2GNN on standard and large-scale graph benchmarks and provide ablations for semantic edge refinement, stable positional encoding, and graph and text fusion. We also add a systematic discussion of recent graph-LLM and graph-MLLM studies to clarify the scope of M2GNN relative to general-purpose multimodal graph LLMs.

The remainder of the paper is structured as follows. Section 2 comprehensively reviews existing methodologies and related research in multimodal fusion for graph-based tasks. Section 3 introduces theoretical foundations, emphasizing multimodal data fusion and its significance in enhancing model performance. Section 4 describes the proposed M2GNN model in detail. Section 5 presents our experimental design, results, and comparative analysis to demonstrate the effectiveness of our approach. Finally, Section 6 summarizes the paper's key findings and outlines directions for future research.

## 2 Related work

The field of multimodal GNNs has advanced rapidly in recent years, driven by the need to improve model performance across various tasks. These networks aim to integrate data from diverse modalities, including text, image, and audio graph structural information, to achieve this objective. Multimodal GNNs integrate image and text data for sophisticated reasoning and question-answering. For instance, the VQA-GNN model integrates unstructured image and textual data with structured knowledge graphs, creating a multimodal semantic graph for passing cross-modal messages and updating node representation (Wang et al., 2023). Multimodal GNNs also integrate multimodal data, such as text and images, to augment the capacity of knowledge graphs to represent information. For example, certain studies have proposed a multimodal knowledge graph embedding method, which combines knowledge graphs with descriptive texts and related images of entities (Liu et al., 2023). This method effectively improves the performance of knowledge graph complementation and relationship prediction tasks (Liu et al., 2023). Moreover, multimodal GNNs enhance the precision of entity recognition by integrating text and structural object characteristics. For example, the GNN-MNER model increases the F1 score on the Twitter dataset using graph attention networks on multimodal interaction graphs to facilitate fine-grained feature fusion and information propagation (Gong et al., 2024). Chen investigated the potential of LLMs for graph machine learning tasks, focusing on node classification. The study proposed two principal pipelines: LLMs-as-Enhancers and LLMs-as-Predictors (Chen et al., 2024). The former uses LLMs to augment the textual attributes of nodes, generating predictions through GNN (Chen et al., 2024). The latter utilizes LLMs directly for prediction. LLMs can enhance the efficacy of text categorization tasks by encoding graph structures into natural language (Chen et al., 2024). Additionally, GraphLLM has been proposed to enhance LLM capabilities in graph inference tasks (Chai et al., 2023). GraphLLM uses a lightweight Transformer encoder-decoder architecture that enables LLMs to understand and interpret graph contexts more effectively by independently processing node information and structural information, thus generating more accurate responses in graph inference tasks (Chai et al., 2023).

Recent studies have increasingly explored the integration of graph learning and large language models (Li et al., 2024). GraphLLM and related graph-LLM methods aim to enhance the graph reasoning ability of LLMs by incorporating graph encoders, graph prompts, or graph-aware instruction tuning (Chai et al., 2023; Wang et al., 2025a). GLEM and similar approaches focus on text-attributed graphs, where language models and GNNs are jointly or collaboratively used for node prediction (Zhao et al., 2023a). These works demonstrate the usefulness of language semantics for graph learning, but their main focus differs from ours: M2GNN is designed for controlled node classification and link prediction rather than general graph reasoning or instruction following.

More recent graph-MLLM studies further extend this direction to multimodal graphs with textual and visual node attributes (Liu et al., 2025; Ye et al., 2025). Graph-MLLM introduces a benchmark for multimodal graph learning and evaluates different ways of using MLLMs as encoders, aligners, or predictors (Liu et al., 2025). MLaGA incorporates textual and visual node attributes together with graph structure through a structure-aware multimodal encoder and multimodal instruction tuning (Fan et al., 2025). Toward Multi-modal Graph Large Language Model discusses a broader framework for general-purpose multimodal graph LLMs across diverse graph data, tasks, and reasoning settings (Wang et al., 2025b). Compared with these works, M2GNN has a narrower scope: it focuses on static graph-text or graph-feature prediction tasks and explicitly studies semantic edge refinement, stable positional encoding, and graph-language prediction fusion for node and link prediction.

Researchers have introduced many open-source big models, including Gemini, LLaMA3, and Microsoft Phi-1, which can be fine-tuned with multimodal capabilities to interpret and respond to diverse content, including text, video, audio, and code (Masalkhi et al., 2024). The model's architectural design and training methodologies emphasize contextual comprehension, rendering it well-suited for numerous potential applications (Huang et al., 2023b; Shi et al., 2024). To further clarify the scope of M2GNN, Table 1 summarizes its relationship to representative recent graph-LLM and graph-MLLM studies.

**Table 1:** Positioning of M2GNN relative to recent graph-LLM and graph-MLLM studies.

Method	Main focus	Modalities	LLM role	Direct NC/LP model?	Difference from M2GNN
GraphLLM	Graph reasoning with LLMs	Graph + text	Graph-aware reasoning and prompting	Partly	M2GNN focuses on controlled node and link prediction rather than general graph reasoning.
GLEM	Text-attributed graph learning	Graph + text	LLM-GNN collaboration	Yes	M2GNN adds explicit semantic edge refinement and dual-stage fusion.
Graph-MLLM	Benchmark for multimodal graph learning	Graph + text + image	MLLM as encoder and predictor	Benchmark-oriented	M2GNN is a task-specific graph-text prediction framework.
MLaGA	Multimodal graph assistant	Graph + text + image	Multimodal instruction tuning	Yes	M2GNN does not target general graph-MLLM instruction following.
Toward MG-LLM	General MG-LLM framework	General multimodal graphs	General-purpose MG-LLM paradigm	No	M2GNN targets static NC/LP tasks.
Graph-augmented LLM Agents	LLM agent systems	Agent graphs, memory and tools	Planning, memory, tool use	No	Complementary, not a direct NC/LP baseline.
M2GNN	Node classification and link prediction	Graph + text features	Edge refinement + semantic predictor	Yes	Controlled graph-text prediction with SPE and adaptive fusion.

However, the oversmoothing problem often arises with deep GNN models. Oversmoothing occurs when representations of different nodes converge to be identical after multiple layers of message passing, reducing the model's discriminative power (Naveed et al., 2023). This issue constrains the deployment of GNNs in deep architectures (Kaddour et al., 2023). Similarly, in opaque LLM models, the internal decision-making process is not readily discernible or amenable to interpretation (Kaddour et al., 2023). The content generated by the models is not always traceable to its logic and rationale, presenting a challenge for applications in sensitive domains such as healthcare and law. LLMs are proficient in numerous tasks; however, their ability to generalize to specific tasks remains limited. Multi-task training results in negative migration between tasks, which affects the learning that occurs in a task-specific manner. Furthermore, the model may experience catastrophic forgetting—whereby previously learned tasks are forgotten—which presents a challenge for applications that require continuous learning and adaptation to new tasks (Kaddour et al., 2023). The performance of LLMs depends on the quality and diversity of the pre-training data (Kaddour et al., 2023), especially if the data is biased (Zhao et al., 2023c). Furthermore, determining the optimal ratio of pre-training data for the model's performance in downstream tasks is crucial (Zhao et al., 2023c). However, the current research in this domain remains inadequate and lacks clear guidance (Naveed et al., 2023; Kaddour et al., 2023; Zhao et al., 2023c).

Data from disparate modalities often have diverse quality, structure, and representation (Zhang et al., 2024). The heterogeneity of these data types challenges the alignment and fusion of cross-modal data (Li and Tang, 2024). For instance, considerable discrepancies exist between images and text formats, which present difficulties for model design and training (Li and Tang, 2024). The literature indicates that heterogeneity requires the consideration of differences across multiple dimensions, including element representation, distribution, structure, information content, and noise (Zhang et al., 2024; Li and Tang, 2024). Data from different modalities can provide complementary information; however, effectively capturing and utilizing these connections remains challenging (Liang et al., 2024). Understanding the connections between modalities from statistical and semantic perspectives can enhance the performance of multimodal models. Regarding multimodal fusion, effective multimodal learning hinges on the ability to align and fuse features from disparate modalities (Liang et al., 2024). Using simple feature splicing in existing methods may prove inadequate for capturing the intricate relationships between modalities (Liang et al., 2024). Conversely, advanced fusion methods, such as attention mechanisms, may increase computational complexity (Stahlschmidt et al., 2022). Furthermore, data from different modalities may introduce noise during collection and processing. For instance, structural noise in images and background noise in audio may affect data quality and model accuracy (Xiaoming et al., 2022).

## 3 Preliminaries

Multimodal Data Fusion (MDF) refers to the integration of data from different modalities to enhance model performance.

Early Fusion: The data from different modalities are fused together in the input stage and then fed into the model for processing. The formula is expressed as Equation 1:


$$\begin{array}{l} \text{z}=f\left({\text{x}}_{1},{\text{x}}_{2},\ldots ,{\text{x}}_{n}\right) \end{array}$$ (1)

where **x**_*i*_ represents the data from the *i*th modality, *f* represents the fusion function, and **z** represents the fused representation.

Late Fusion: In the decision stage, the predictions from different modalities are integrated. The formula is expressed as follows (Equation 2):


$$\begin{array}{l} \text{y}=g\left(h_{1}\left({\text{x}}_{1}\right),h_{2}\left({\text{x}}_{2}\right),\ldots ,h_{n}\left({\text{x}}_{n}\right)\right) \end{array}$$ (2)

where *h*_*i*_ represents the model processing the *i*th modality's data, *g* represents the fusion function, and **y** represents the final prediction.

Multimodal Representation Learning (MRL) is to identify and represent the associations between different modalities. This is achieved by learning a common representation.

Aligned Representation: The features of different modalities are aligned in a semantic sense in order to facilitate cross-modal understanding and manipulation through the use of alignment techniques. The formula is expressed as follows (Equation 3):


$$\begin{array}{l} L_{align}=\underset{i,j}{\sum }d\left({\text{z}}_{i},{\text{z}}_{j}\right) \end{array}$$ (3)

where $L_{align}$ is the alignment loss, and *d* is a distance function measuring the difference between representations from different modalities.

Graph-Structured Data: A graph is represented as a tuple G=(V,A,T). Here, V denotes a set of N=|V| nodes, and $A\in {\mathbb{R}}^{N\times N}$ is the adjacency matrix that describes the connections between nodes. Additionally, $t_{n}\in T$ represents the textual data associated with each node n∈V in graph G, which consists of a sequence of *L*_*n*_ language tokens.

Problem Statement: Given the observed graph G=(V,A,T) with noisy structural information, our objective is to extend multimodal information, improve multimodal methods, and integrate predictor results. This involves extending and integrating node and edge information to uncover hidden relationships among nodes. By refining the original adjacency matrix A and obtaining a more informative graph structure $\tilde{A}$, we can more accurately capture the underlying node dependencies, resulting in an updated graph $\tilde{G}=\left(V,\tilde{A}\right)$. This refinement process deepens our understanding of the graph structure, thereby enhancing the performance of downstream tasks by leveraging the updated graph structure $\tilde{G}=\left(V,\tilde{A}\right)$.

## 4 Methodology

**Figure 2:**
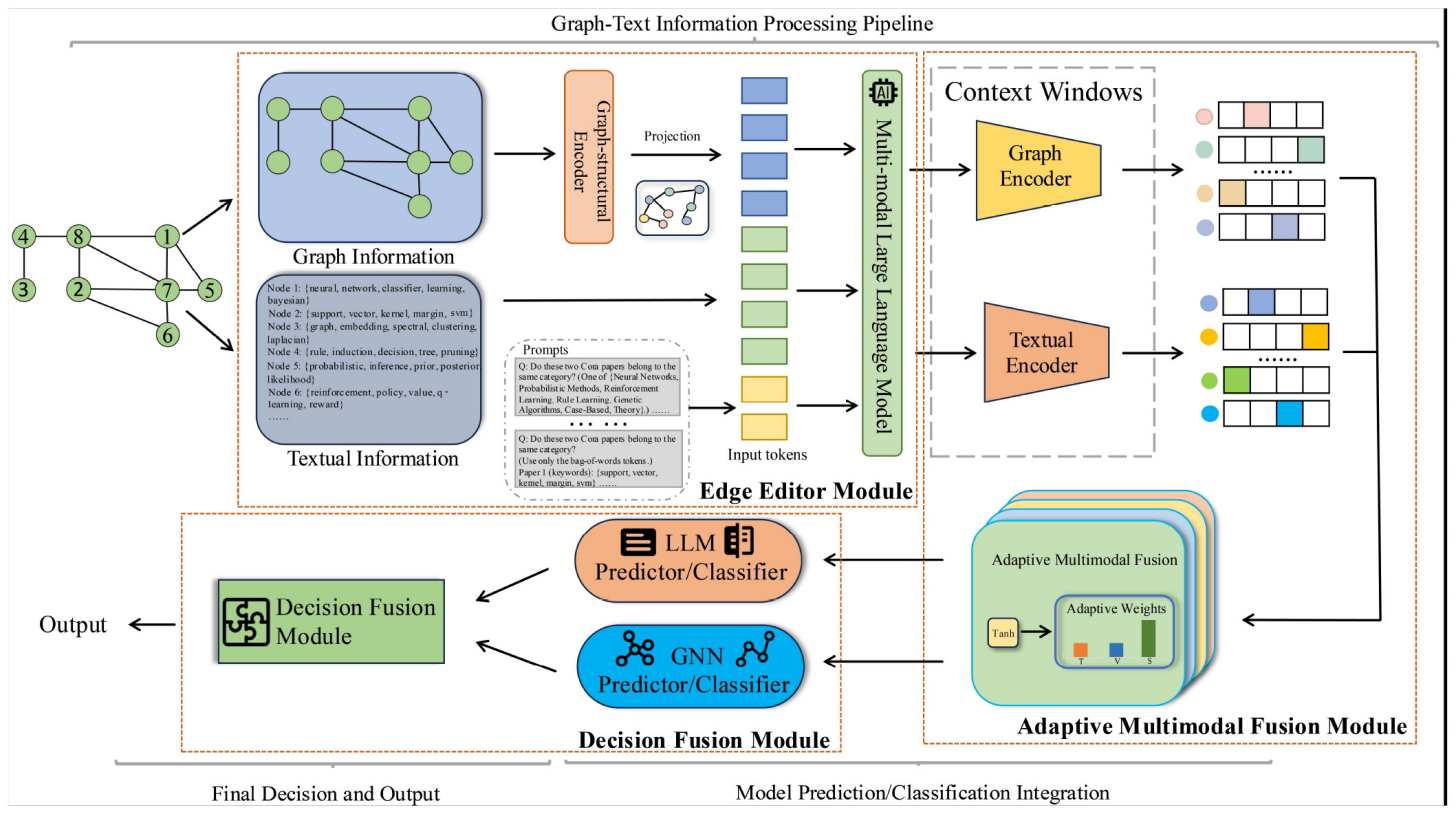
Overview of the M2GNN framework: a unified pipeline for graph-text representation and multimodal adaptive learning.

This section introduces the proposed M2GNN model in detail. First, the instruction-tuning process of LLM is described to demonstrate its adaption to graph-based tasks. Second, the multimodal fusion approach is explained, illustrating how LLM and GNN features are integrated for improved representation learning. Third, the dual-predictor framework combining LLM-based and GNN-based predictors is introduced to enhance classification and link prediction performance. Finally, a benchmark statistical analysis is provided to evaluate the effectiveness of M2GNN across multiple datasets. Figure 2 depicts the overall workflow of the M2GNN model, highlighting the interactions between these key components. Although recent graph-MLLMs pursue general multimodal graph reasoning, M2GNN deliberately keeps a message-passing graph encoder because the target tasks are node classification and link prediction on static attributed graphs. This design allows the model to preserve the inductive bias of GNNs for local topology while using LLM-derived semantics as complementary information. The goal is not to replace graph foundation models, but to provide a lightweight and resource-aware graph–LLM pipeline for discriminative prediction tasks.

### 4.1 LLM-assisted semantic encoding and edge refinement

We use LLM-derived semantic embeddings for candidate-restricted edge refinement. Directly evaluating all possible node pairs would require *O*(*N*^2^) pair scoring and is infeasible for large graphs. Therefore, M2GNN does not perform all-pair LLM inference. Instead, each node obtains a semantic embedding once through an offline LLM and text encoder, and these embeddings are cached:

LLM input serialization and prompt design. For each node vi, we construct a label-free textual input si using a fixed serialization template. The input contains four fields: (i) a task-level instruction, (ii) dataset and node metadata, (iii) node textual attributes such as title, abstract, keywords, or bag-of-words tokens, and (iv) a restricted local structural summary including the node degree and sampled one-hop/two-hop neighbor keywords. Ground-truth labels of validation and test nodes are never included in the prompt. For datasets without natural-language node descriptions, we do not generate artificial labels through the LLM; instead, the original numeric node features are projected into the same semantic space by a trainable feature projector.


$$\begin{array}{l} h_{i}=\text{LLM}\left(s_{i}\right),\;i\in V, \end{array}$$ (4)

In Equation 4, LLM(·) specifically refers to embedding extraction rather than free-form text generation. The returned vector is normalized and projected to the same dimension as the graph representation before fusion.

For each node *v*_*i*_, we construct a sparse candidate set $C_{i}$ using existing 2-hop neighbors and approximate nearest-neighbor retrieval in the cached semantic embedding space (Equation 5):


$$\begin{array}{l} C_{i}={\text{TopK}}_{j\neq i}\left(\text{sim}\left(h_{i},h_{j}\right)\right)\cup N_{2hop}\left(i\right),\;|C_{i}|\leq k. \end{array}$$ (5)

The edge predictor is applied only to candidate pairs $\left(i,j\right)\in C=\cup_{i}C_{i}$, rather than to all *N*^2^ pairs (Equation 6):


$$\begin{array}{l} {\hat{y}}_{ij}=\eta \left(\left[h_{i}\|h_{j}\right]\right),\;\left(i,j\right)\in C. \end{array}$$ (6)

The edge predictor is trained with the binary cross-entropy loss (Equation 7):


$$\begin{array}{l} L_{edge}=-\frac{1}{\left|P\right|}\underset{\left(i,j\right)\in P}{\sum }\left[y_{ij}\log{\hat{y}}_{ij}+\left(1-y_{ij}\right)\log\left(1-{\hat{y}}_{ij}\right)\right], \end{array}$$ (7)

where P denotes the sampled node-pair set and ŷ_*ij*_ is the predicted edge probability.

The accepted edge edits are selected by a confidence threshold τ and an edit budget *r* (Equation 8):


$$\begin{array}{l} \Delta E=\left\{\left(i,j\right)\in C:{\hat{y}}_{ij}\geq \tau \right\},\;|\Delta E_{i}|\leq r. \end{array}$$ (8)

The refined adjacency is then constructed by applying the accepted sparse edits to the original graph (Equation 9):


$$\begin{array}{l} \hat{A}=A⊕\Delta A, \end{array}$$ (9)

where ⊕ denotes sparse edge addition or deletion. This formulation avoids prompting the LLM with the full adjacency matrix and avoids dense *O*(*N*^2^) edge inference. The edge-scoring cost is *O*(*kNd*), and constructing the refined sparse graph costs *O*(*M* + *kN*).

To avoid unconstrained LLM-driven graph modification, we restrict the accepted edge edits by an edit budget and a spectral proximity constraint (Equation 10):


$$\begin{array}{l} \|\hat{A}-A{\|}_{0}\leq rN,\;\|L\left(\hat{A}\right)-L\left(A\right){\|}_{F}\leq \epsilon , \end{array}$$ (10)

where *r* is the maximum average number of accepted edits per node and ϵ controls the allowed perturbation of the normalized graph Laplacian. This constraint does not provide a global convergence guarantee for arbitrary noisy graphs, but it ensures that subsequent graph encoding is performed on a bounded perturbation of the original graph.

### 4.2 Stable graph position encoding

Positional encoding is performed in graph-structured data to furnish each node with information regarding its relative position within the graph, enhancing their discernibility. Common methodologies include using eigenvalues and eigenvectors of the graph Laplacian matrix as positional encoding, which capture global structural information about the graph. However, traditional Laplacian feature-based encoding methods face two main challenges. Firstly, the same Laplacian matrix can have multiple feature decompositions, known as non-uniqueness. Secondly, small perturbations to the Laplace matrix can result in entirely different feature spaces, leading to instability in position coding. To address these issues, we adopt a stable positional encoding strategy to improve the robustness of structural representations after semantic graph refinement.

The Stable and Expressive Positional Encodings (SPE) method achieves positional encoding stability by avoiding the complex division of feature subspace (Huang et al., 2023a). The key lies in using eigenvalues to weight and sum the eigenvectors, achieving “soft partitioning.”

Firstly, the location coding information is extracted from the manipulated graph. Then, the Laplace matrix decomposition of the graph is performed, and its normalized Laplace matrix is defined as follows:


$$\begin{array}{l} {\hat{D}}_{ii}=\underset{j}{\sum }{\hat{A}}_{ij},\;\hat{L}=I-{\hat{D}}^{-1/2}\hat{A}{\hat{D}}^{-1/2}. \end{array}$$ (11)

Performing eigenvalue decomposition on the Laplacian matrix *L*, we obtain the eigenvalues Λ and the eigenvectors *V*:


$$\begin{array}{l} L=V\Lambda V^{⊤} \end{array}$$ (12)

Permutation invariant functions are a class of functions whose output is insensitive to changes in the alignment of the input data. In the context of eigenvector processing of graphs, the function is invariant to base transformations (e.g., orthogonal transformations of eigenvectors) when processing eigenvectors.

We partition the eigenvalues, apply a permutation invariant function to each interval, and apply multiple monotonic functions φ_*i*_ to eigenvalue matrix Λ. We apply the function φ to the eigenvalue λ, generating a new vector, and then compute the result together with the eigenvector *V*:


$$\begin{array}{l} {\left[\varphi_{\ell }\left(\lambda \right)\right]}_{i}=\text{spline}\left(\lambda_{i}\right) \end{array}$$ (13)

where spline is a piecewise polynomial function applied to the eigenvalues.

Stability of SPE under bounded graph perturbation. Let *L* = *L*(*A*) and $\hat{L}=L\left(\hat{A}\right)$ be the normalized Laplacians before and after edge refinement. Under the spectral proximity constraint in Equation 10, we have $\|\hat{L}-L{\|}_{2}\leq \|\hat{L}-L{\|}_{F}\leq \epsilon$. Let *V*_*d*_ and ${\hat{V}}_{d}$ denote the eigenspaces associated with the first *d* eigenvalues, and let γ > 0 be the eigengap between the retained eigenspace and its complement. Standard spectral perturbation results imply:


$$\begin{array}{l} \|\sin\Theta \left({\hat{V}}_{d},V_{d}\right){\|}_{2}\leq \frac{\epsilon }{\gamma }. \end{array}$$ (14)

Moreover, SPE uses expressions of the form *V*diag(ϕ(λ))*V*^⊤^, which are invariant to eigenvector sign flips and orthogonal basis rotations within the same eigenspace. If ϕ and ρ are Lipschitz-continuous with bounded constants, the resulting positional encodings satisfy:


$$\begin{array}{l} \|Z\left(\hat{V},\hat{\lambda }\right)-Z\left(V,\lambda \right){\|}_{F}\leq C_{\text{SPE}}\frac{\epsilon }{\gamma }, \end{array}$$ (15)

where *C*_SPE_ depends on the Lipschitz constants of ϕ and ρ. Therefore, SPE is stable when the accepted graph refinement is small and the retained eigenspace is sufficiently separated. This bound also clarifies the limitation: SPE may become less stable when the eigengap is very small or when graph editing introduces large spectral perturbations.

Given the first *d* smallest eigenvalues λ ∈ ℝ^*d*^ of the Laplacian matrix and the corresponding eigenvector matrix *V* ∈ ℝ^*n*×*d*^, we apply the monotonic functions φ_ℓ_ and ρ for weighted summation. The formula is as follows:


$$\begin{array}{l} \begin{array}{l} \text{Z}\left(V,\lambda \right)=\rho (V\cdot \text{diag}\left(\varphi_{1}\left(\lambda \right)\right)\cdot V^{⊤},\\ \;V\cdot \text{diag}\left(\varphi_{2}\left(\lambda \right)\right)\cdot V^{⊤},\\ \;\ldots ,\\ \;V\cdot \text{diag}\left(\varphi_{m}\left(\lambda \right)\right)\cdot V^{⊤}) \end{array} \end{array}$$ (16)

where *V* ∈ ℝ^*n*×*d*^ is the eigenvector matrix, λ ∈ ℝ^*d*^ represents the corresponding eigenvalue vector; $\varphi_{\ell }:{\mathbb{R}}^{d}\to {\mathbb{R}}^{d}$ is a monotonic function (such as DeepSets or MLP); ρ : ℝ^*n*×*n*×*m*^ → ℝ^*n*×*p*^ is a monotonic neural network used to generate the final node position encoding; diag(φ_ℓ_(λ)) denotes the diagonal matrix obtained by applying the function φ_ℓ_ to the eigenvalue vector.

For scalability, we do not compute the full eigendecomposition of the *N* × *N* Laplacian. Instead, we compute only the first *K* eigenpairs using a sparse eigensolver such as Lanczos or LOBPCG, where *K* ≪ *N*. For sparse graphs, this gives an approximate cost of *O*(*sKM*), where *s* is the number of solver iterations, and memory cost *O*(*M* + *KN*). This avoids dense *O*(*N*^2^) storage and dense *O*(*N*^3^) eigendecomposition.


$$\begin{array}{l} h_{i}^{G}=\text{MultiHead}(Q,K,V)=\text{Concat}(\;{\text{head}}_{1},\\ \;{\text{head}}_{2},\\ \;\ldots ,\\ \;{\text{head}}_{h})W^{O} \end{array}$$ (17)

where *Q, K*, and *V* represent the query, key, and value, respectively, *W*^*O*^ is the output weight matrix, and *h* denotes the number of attention heads. Each attention head is computed as follows:


$$\begin{array}{l} {\text{head}}_{i}=\text{Attention}\left(QW_{i}^{Q},KW_{i}^{K},VW_{i}^{V}\right) \end{array}$$ (18)

The computation of the attention mechanism is as follows:


$$\begin{array}{l} \text{Attention}\left(Q,K,V\right)=\text{softmax}\left(\frac{QK^{T}}{\sqrt{d_{k}}}\right)V \end{array}$$ (19)

where $Q=HW_{i}^{Q},K=HW_{i}^{K},V=HW_{i}^{V}$, and *d*_*k*_ is the dimension of the key. The similarity between the query and key is calculated using dot product, and then softmax is applied for normalization, followed by weighted sum to obtain the values.

### 4.3 Adaptive multimodal fusion module

We propose an adaptive multimodal fusion module that dynamically balances the contributions of each modality on a per-node basis to integrate heterogeneous information from the graph structure and textual semantics. Given a node *v*_*i*_, we first obtain two modality-specific representations: the graph-based representation ${\text{h}}_{i}^{G}\in {\mathbb{R}}^{d}$ extracted from the graph encoder, and the text-based representation ${\text{h}}_{i}^{T}\in {\mathbb{R}}^{d}$ derived from the textual encoder. These two embeddings capture structural and semantic information, respectively.

To adaptively control the fusion of these modalities, we introduce a lightweight weight generation network to learn the relative importance of each modality for each node. Specifically, the intermediate attention scores for the graph and text representations are computed as follows:


$$\begin{array}{l} \begin{array}{c} \alpha_{i}^{G}={\text{w}}_{G}^{⊤}\tanh\left({\text{W}}_{G}{\text{h}}_{i}^{G}+{\text{b}}_{G}\right)\\ \alpha_{i}^{T}={\text{w}}_{T}^{⊤}\tanh\left({\text{W}}_{T}{\text{h}}_{i}^{T}+{\text{b}}_{T}\right) \end{array} \end{array}$$ (20)

where ${\text{W}}_{G},{\text{W}}_{T}\in {\mathbb{R}}^{d^{\prime }\times d}$, ${\text{w}}_{G},{\text{w}}_{T}\in {\mathbb{R}}^{d^{\prime }}$, and ${\text{b}}_{G},{\text{b}}_{T}\in {\mathbb{R}}^{d^{\prime }}$ are learnable parameters. These scores are then normalized using a softmax function to produce the final adaptive weights:


$$\begin{array}{l} \begin{array}{c} w_{i}^{G}=\frac{\exp\left(\alpha_{i}^{G}\right)}{\exp\left(\alpha_{i}^{G}\right)+\exp\left(\alpha_{i}^{T}\right)}\\ w_{i}^{T}=\frac{\exp\left(\alpha_{i}^{T}\right)}{\exp\left(\alpha_{i}^{G}\right)+\exp\left(\alpha_{i}^{T}\right)} \end{array} \end{array}$$ (21)

Using these weights, the fused representation ${\text{h}}_{i}^{\text{fused}}$ is computed as a convex combination of the two modality-specific features:


$$\begin{array}{l} {\text{h}}_{i}^{\text{fused}}=w_{i}^{G}\cdot {\text{h}}_{i}^{G}+w_{i}^{T}\cdot {\text{h}}_{i}^{T}. \end{array}$$ (22)

The resulting fused representation effectively captures structural and semantic contextual cues, with modality importance dynamically adjusted per instance. This adaptive fusion strategy enhances the robustness of the model against noisy or missing modality information and improves generalization across diverse graph-based scenarios.

In Equation 22, both ${\text{h}}_{i}^{G}$ and ${\text{h}}_{i}^{T}$ are d-dimensional vectors. The graph representation ${\text{h}}_{i}^{G}$ is obtained from the refined graph encoder after stable positional encoding, whereas ${\text{h}}_{i}^{T}$ is the projected LLM-derived semantic representation defined in Equation 4. Before fusion, both representations are passed through layer normalization to reduce scale mismatch between graph and language modalities. The adaptive weights $w_{i}^{G}$ and $w_{i}^{T}$ are node-specific and satisfy $w_{i}^{G}$ + $w_{i}^{T}$ = 1. During training, gradients are backpropagated through the graph encoder, the text projection layer, and the fusion network. For closed-source API-based LLMs, the cached LLM embeddings are treated as fixed input features and are not updated. For local open-source LLMs, only the LoRA adapters and downstream prediction heads are trainable. This implementation makes the fusion step reproducible and avoids repeated LLM inference during every training epoch.

The feature-level fusion in Equation 22 is designed to construct a unified node representation before the final prediction stage. At this stage, the graph-derived representation $h_{i}^{G}$ captures local topology and structural dependencies, whereas the text-derived representation $h_{i}^{T}$ provides semantic information from node attributes and LLM-derived embeddings. The adaptive weights $w_{i}^{G}$ and $w_{i}^{T}$ allow the model to balance these two modalities at the representation level, thereby reducing the effect of noisy or incomplete graph/text features before prediction.

### 4.4 LLM-based and GNN classifier and predictor

Given the fused node representation ${\text{h}}_{i}^{\text{fused}}$ derived from the adaptive multimodal fusion module (Equation 22), we introduce two complementary classification and prediction mechanisms: an LLM-based predictor and a GNN-based predictor.

For open-source or locally fine-tuned LLM backbones, the semantic predictor is implemented as a Transformer encoder followed by a trainable classification head:


$$\begin{array}{l} u_{i}=f_{\text{LLM}}\left(E_{i}\right),\;z_{\text{LLM}}=W_{\text{LLM}}u_{i}+b_{\text{LLM}}. \end{array}$$ (23)

For closed-source API-based LLMs, we use deterministic zero-temperature inference and do not update *W*_*LLM*_ or *b*_*LLM*_. These API-based results are reported separately as LLM-backbone analysis rather than as trainable end-to-end baselines.

Simultaneously, the GNN-based classifier exploits a message-passing approach explicitly structured to capture topological and relational patterns within graph-structured data. Using ${\text{h}}_{i}^{\text{fused}}$ as initial node features, the node embeddings are iteratively refined through multiple graph convolutional layers. Formally, for the (*k* + 1)-th layer, the node representation ${\text{h}}_{i}^{\left(k+1\right)}$ is updated as follows:


$$\begin{array}{l} {\text{h}}_{i}^{\left(k+1\right)}=\sigma \left({\text{W}}^{\left(k\right)}\left(\underset{j\in N\left(i\right)}{\sum }\frac{1}{c_{ij}}{\text{h}}_{j}^{\left(k\right)}\right)+{\text{b}}^{\left(k\right)}\right) \end{array}$$ (24)

where N(i) indicates the neighbors of node *i*, **W**^(*k*)^ and **b**^(*k*)^ denote learnable parameters, *c*_*ij*_ is a normalization coefficient, and σ(·) is an activation function such as ReLU. After propagating through *K* layers, the resulting node embeddings ${\text{H}}_{i}^{\text{GNN}}={\text{h}}_{i}^{\left(K\right)}$ are processed by a fully connected layer to generate the GNN-based logits **z**_GNN_:


$$\begin{array}{l} {\text{z}}_{\text{GNN}}={\text{W}}_{\text{GNN}}{\text{H}}_{i}^{\text{GNN}}+{\text{b}}_{\text{GNN}} \end{array}$$ (25)

where **W**_GNN_ and **b**_GNN_ are learnable parameters associated with the GNN classification layer.

Finally, the logits **z**_LLM_ and **z**_GNN_ produced by these distinct yet complementary classifiers are integrated through the dynamic fusion approach described in Section 4.5. This integration ensures robust predictions by leveraging semantic and structural representations inherent to the input data.

### 4.5 Dynamic fusion via attention-style gating

We introduce a dynamic fusion mechanism inspired by attention-style gating to integrate the complementary strengths of the GNN-based structural encoder and the LLM-based semantic encoder. Rather than adopting static or manually-tuned fusion coefficients, our model learns to adaptively determine the importance of each modality per instance.

Let ${\text{z}}_{\text{LLM}}\in {\mathbb{R}}^{d}$ and ${\text{z}}_{\text{GNN}}\in {\mathbb{R}}^{d}$ denote the logits (pre-softmax outputs) from the LLM and GNN classifiers, respectively. These are concatenated to form a joint representation:


$$\begin{array}{l} {\text{z}}_{\text{concat}}=\left[{\text{z}}_{\text{LLM}};{\text{z}}_{\text{GNN}}\right]\in {\mathbb{R}}^{2d}. \end{array}$$ (26)

We feed this joint vector into a lightweight two-layer feed-forward gating network to compute a fusion coefficient α ∈ (0, 1):


$$\begin{array}{l} \text{h}=\text{ReLU}\left(W_{1}{\text{z}}_{\text{concat}}+{\text{b}}_{1}\right), \end{array}$$ (27)

$$\begin{array}{l} \alpha =\sigma \left(W_{2}\text{h}+{\text{b}}_{2}\right), \end{array}$$ (28)

where σ(·) denotes the sigmoid activation function to ensure α is bounded between 0 and 1. For simplicity, we use a scalar α to control the fusion globally across all classes, though this can be extended to a vector for per-class weighting.

The final prediction ${\text{y}}_{\text{fused}}\in {\mathbb{R}}^{d}$ is obtained via a convex combination of the softmax-normalized predictions from the two modalities:


$$\begin{array}{l} {\text{y}}_{\text{fused}}=\alpha \cdot \text{Softmax}\left({\text{z}}_{\text{LLM}}\right)+\left(1-\alpha \right)\cdot \text{Softmax}\left({\text{z}}_{\text{GNN}}\right). \end{array}$$ (29)

The prediction-level fusion in Equation 29 serves a different purpose from the feature-level fusion in Equation 22. While Equation 22 combines graph and text features into a shared node representation, Equation 29 combines the outputs of two predictors with different inductive biases. The GNN-based predictor is more reliable when neighborhood structure is informative, whereas the LLM-based predictor is more useful when textual semantics provide stronger evidence. The gating coefficient α therefore acts as a decision-level reliability estimator, allowing the final prediction to adaptively rely on the more trustworthy branch for each instance. This dual-stage design separates multimodal representation learning from final prediction calibration. Accordingly, in the ablation study, modality-removal variants are used to analyze the feature-level fusion stage in Equation 22, whereas branch-removal variants are used to analyze the prediction-level fusion stage in Equation 29.

Because *y*_*fused*_ is a convex combination of two probability distributions, the fusion step is non-expansive with respect to branch prediction errors. Let *p*_*LLM*_ = Softmax(*z*_*LLM*_), *p*_*GNN*_ = Softmax(*z*_*GNN*_), and *y* be the target distribution. Then (Equation 30):


$$\begin{array}{l} \|y_{fused}-y{\|}_{1}\leq \alpha \|p_{LLM}-y{\|}_{1}+\left(1-\alpha \right)\|p_{GNN}-y{\|}_{1}. \end{array}$$ (30)

Thus, the fused prediction error is bounded by the weighted errors of the semantic and structural branches. In sparse graph regimes, where the GNN branch may be less reliable because of limited neighborhood information, the gate can assign more weight to the LLM branch; conversely, when textual information is noisy or incomplete, the gate can rely more on the GNN branch. This bound does not guarantee that fusion always outperforms both branches, but it shows that the fusion step itself does not amplify prediction errors beyond the weighted branch errors.

### 4.6 M2GNN architecture

The proposed M2GNN model integrates multimodal information by seamlessly combining the complementary capabilities of GNNs and LLMs. Initially, M2GNN uses LLM-generated node embeddings to predict and refine edge structures, strategically enhancing graph connectivity through targeted addition and deletion of edges. Subsequently, the optimized graph structure undergoes eigen-decomposition to produce stable positional encodings, which are further refined using multi-head attention mechanisms to derive graph embeddings.

Concurrently, textual features from node descriptions are independently encoded, capturing semantic context through a Transformer-based LLM encoder. An adaptive multimodal fusion mechanism then dynamically weights these graph-derived and text-derived representations, ensuring robust performance even with missing or noisy inputs. Finally, a dual-predictor framework combines predictions from a semantic-focused Transformer classifier and a structurally aware GNN classifier via an attention-inspired gating network, dynamically determining the optimal balance of semantic and structural information for accurate and resilient graph-based predictions. The complete operational flow of M2GNN is systematically illustrated in Algorithm 1.

Algorithm 1 M2GNN end-to-end processing pipeline. 
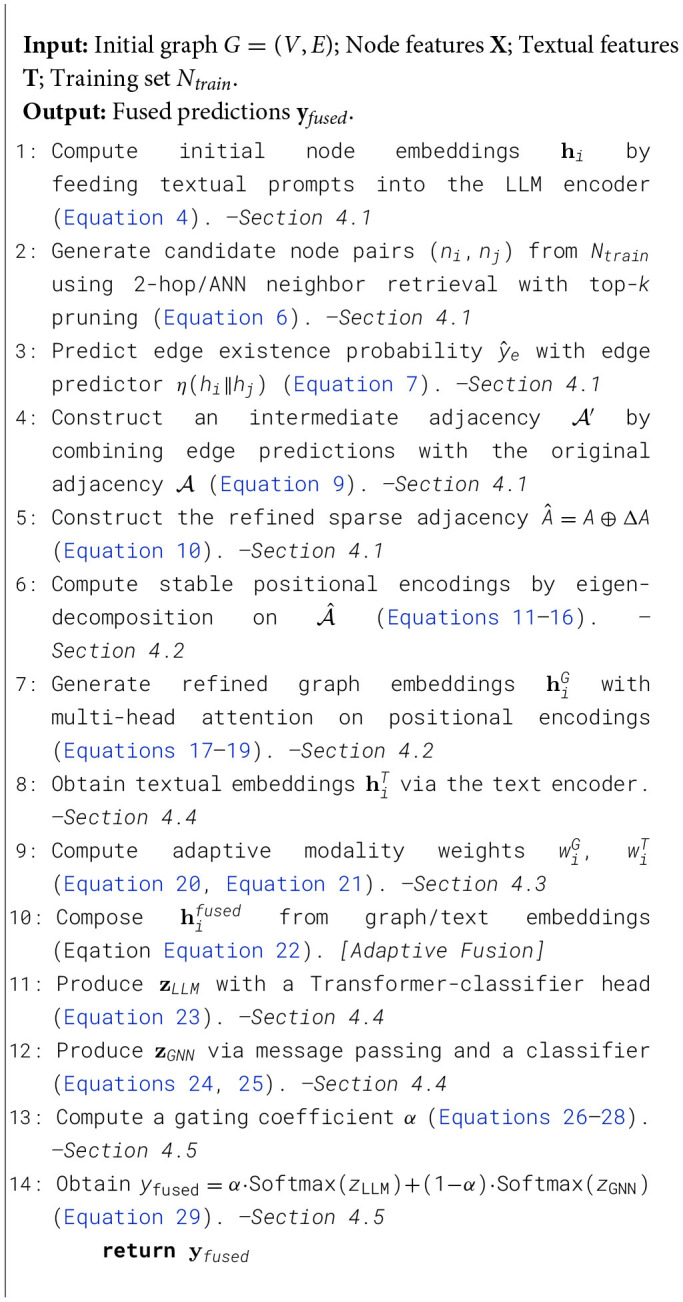

### 4.7 Complexity and resource analysis

We next discuss the computational complexity and resource usage of M2GNN. Let *N* = |*V*| and *M* = |*E*| denote the number of nodes and edges in a graph, and let *d* be the hidden dimension, *K* the number of eigenvectors used for positional encodings, *L* the number of GNN layers, and *H* the number of attention heads.

LLM-guided structural refinement: In the first stage, we obtain node-level semantic embeddings *h*_*i*_ = LLM(*s*_*i*_) for all *i* ∈ *V* (Equation 4). If we treat a single LLM call as having cost *C*_LLM_, the overall complexity of this step is $O\left(N\cdot C_{\text{LLM}}\right)$. In practice, these calls are performed once as an offline pre-processing step and cached for all subsequent training and evaluation runs. The lightweight edge predictor η(*h*_*i*_∥*h*_*j*_) operates on a sparse set of candidate node pairs obtained by 2-hop or approximate nearest neighbor retrieval (Equation 6), resulting in O(kNd) computations where *k* ≪ *N* is the maximum number of candidates per node. Constructing the intermediate adjacency *A*′ (Equation 9) and refining it into Â under a spectral proximity budget (Equation 10) are both linear in the number of edited edges and thus scale near-linearly with *M* in our implementation.

Stable positional encoding and graph encoder: The SPE module operates on the normalized Laplacian *L* of the refined adjacency Â (Equations 11–16). We rely on sparse eigensolvers to compute the first *K* eigenpairs, leading to a complexity that is approximately O(KM) for large sparse graphs. The subsequent multi-head attention over positional encodings (Equations 17–19 requires O(HMd) operations, as each head performs attention over local neighborhoods. The GNN encoder then applies *L* layers of message passing (Equation 24, each scaling as O(Md), resulting in an overall cost of O(LMd) for the structural branch.

Text encoder, fusion and prediction heads: The textual encoder produces semantic embeddings $h_{i}^{T}$ (Algorithm 1, step 8). When implemented with a frozen LLM or a smaller transformer, this cost is $O\left(N\cdot C_{\text{text}}\right)$ with *C*_text_ ≪ *C*_LLM_, and can likewise be pre-computed and cached. The adaptive fusion module (Equations 20–22 uses a small multi-layer perceptron to compute modality weights and a weighted sum of $h_{i}^{G}$ and $h_{i}^{T}$, which incurs $O\left(Nd^{2}\right)$ operations but is typically dominated by the graph encoder. The LLM-based classifier and GNN-based classifier (Equations 23–25, together with the gating network (Equations 26–28, add only $O\left(Nd^{2}\right)$ computations and negligible memory overhead compared to the graph convolution and eigendecomposition stages.

Empirical scalability: On smaller citation graphs such as Cora and Citeseer, M2GNN runs within seconds per epoch on a single GPU. On larger static graphs such as DBLP(Aminer), OGBN-Arxiv, and Reddit, the main computational overhead comes from three sources: offline LLM embedding and semantic edge scoring, sparse eigensolver-based SPE computation, and message passing on the refined graph. Since the LLM-derived embeddings and candidate edge scores are cached, they are not recomputed at every epoch. Table 2 provides the measured runtime and memory footprint. These results indicate that M2GNN is computationally heavier than pure GNN baselines but remains practical for the evaluated large-scale static graph benchmarks under cached LLM pre processing. To complement the asymptotic complexity analysis, we further report practical runtime and memory usage on the large-scale datasets. Since closed-source LLM APIs do not expose model parameters or computational graphs, we do not estimate their FLOPs directly. Instead, we measure wall-clock runtime, one-time LLM cache preprocessing time, training time per epoch, total training time, inference time, and peak GPU memory. This protocol better reflects the practical cost of using M2GNN, because LLM-derived semantic embeddings and candidate edge scores are computed once offline and then cached for all subsequent training and evaluation runs.

**Table 2:** Runtime and memory comparison on large-scale graph datasets.

Dataset	Method	Offline LLM/cache time	SPE/graph refinement time	Train time/epoch	Total training time	Inference time	Peak GPU memory
DBLP (Aminer)	GraphEdit	–	125 min 5 s	15 s	25 min 10 s	2.5 s	19.8 GB
DBLP (Aminer)	GLEM-GNN	–	–	12 s	20 min 05 s	1.8 s	18.2 GB
DBLP (Aminer)	M2GNN	380 min	45 min	18 s	30 min 15 s	2.8 s	22.5 GB
OGBN-Arxiv	GraphEdit	–	18 min 45 s	3.5 s	5 min 50 s	0.8 s	12.4 GB
OGBN-Arxiv	GLEM-GNN	–	–	2.8 s	4 min 40 s	0.5 s	10.6 GB
OGBN-Arxiv	M2GNN	45 min	8 min	4.2 s	7 min 00 s	1.1 s	14.8 GB
Reddit	GraphSAGE	–	–	8.5 s	14 min 10 s	1.5 s	15.3 GB
Reddit	GraphEdit	–	55 min 20 s	12.5 s	20 min 50 s	2.2 s	20.1 GB
Reddit	M2GNN	65 min	25 min	14.2 s	23 min 40 s	2.5 s	21.6 GB

We report wall-clock time and peak GPU memory under the same hardware platform. For M2GNN, offline LLM cache time denotes one-time semantic embedding extraction and candidate edge scoring. Cached LLM-derived embeddings are reused during training and inference, so LLM inference is not repeated at every epoch.

The results show that M2GNN introduces additional one-time preprocessing cost due to LLM-based semantic embedding and candidate edge scoring. However, after caching these semantic representations, the repeated training and inference stages are dominated by sparse graph encoding, SPE computation, and message passing. On large-scale datasets such as DBLP(Aminer), OGBN-Arxiv, and Reddit, M2GNN remains trainable on RTX 4090 GPUs, with peak memory consumption within the available GPU memory budget. Therefore, the practical cost of M2GNN should be interpreted as a combination of offline semantic preprocessing and online graph prediction, rather than as repeated LLM inference during every training epoch.

## 5 Experiments

In this section, we empirically evaluate the proposed M2GNN model.

### 5.1 Experimental settings

Datasets: In this study, we use several benchmark datasets widely used in graph neural network research, including Cora, Pubmed, and CiteSeer, as well as datasets from the Open Graph Benchmark (OGB) and DBLP from Aminer. The characteristics of the datasets are listed in Table 3. The following is a brief description of these datasets:

**Table 3:** Statistics of Experimental Datasets.

Dataset	# Nodes	# Edges	# Feat
Cora	2,708	5,429	1,433
Citeseer	3,186	3,703	4,277
PubMed	19,717	44,335	500
PPI	56,944	818,716	50
DBLP (Aminer)	1,572,277	2,084,019	1,903
OGBN-Arxiv	169,343	1,166,243	128
Reddit	232,965	11,606,919	602

Cora: The Cora dataset contains 2,708 papers in the field of machine learning, which are categorized into seven categories. Nodes represent papers and edges represent citation relationships between papers. Each node is represented by a set of word vectors with a total of 1,433 independent lexical features.

Pubmed: The Pubmed dataset is a much larger citation network containing 19,717 papers in the field of biomedicine, each represented by a 500-dimensional TF-IDF word vector. These papers are categorized into three classes, with edges between nodes indicating citation relationships.

CiteSeer: The Citeseer dataset contains 3,186 papers and 3,703 citation links. Each node is represented by a 4,277-dimensional bag-of-words feature vector, and the papers are classified into six categories.

PPI: The Protein–Protein Interaction (PPI) benchmark consists of multiple graphs (56,944 nodes and 818,716 edges in total), where nodes are proteins and edges indicate physical interactions. Each protein is described by a 50-dimensional feature vector and annotated with multiple Gene Ontology terms (121 labels in total). Following common practice, we evaluate with micro-F1.

DBLP (Aminer): This citation network includes 1,572,277 academic papers and 2,084,019 citation links. Each paper is encoded using a 1,903-dimensional feature vector, typically constructed from its title and abstract. The task is to determine the research area of each paper from a set of predefined domains.

OGBN-ArXiv: This dataset comprises 169,343 nodes and 1,166,243 edges, where each node corresponds to an academic paper and edges indicate citation links between them. Node features are 128-dimensional vectors derived from the embeddings of paper titles and abstracts. The goal is to classify papers into one of 40 distinct research categories.

Reddit: The Reddit dataset contains 232,965 posts with 11.6M edges capturing user–post interaction co-occurrence. Nodes correspond to posts and labels correspond to 41 subreddit communities. Node features are 602-dimensional text/interaction descriptors. We follow the original GraphSAGE split.

Experimental scope. The evaluated datasets cover standard citation graphs and larger static attributed graphs, including DBLP (Aminer), OGBN-Arxiv, and Reddit. We emphasize that M2GNN is designed for static attributed graphs with textual or feature-based node information. It is not formulated as a temporal graph model or a relation-specific knowledge graph completion model. Therefore, massive knowledge graphs such as Freebase-style KG subsets and dynamic graph benchmarks require different relation-aware or temporal objectives and are outside the scope of the present study. We have revised the claims accordingly and report these directions as limitations rather than claiming generality to billion-scale or dynamic graph settings. The present study evaluates static attributed graphs up to the million-node scale under cached LLM preprocessing and sparse candidate refinement.

Benchmark Models: In order to conduct a comprehensive evaluation of the performance of M2GNN models in benchmarking, a variety of open and closed source models were selected for analysis. Specifically, the zero-shot performance of the latest closed-source LLMs, including ChatGPT-4.5 (OpenAI, 2025) and ChatGPT-4o (Hurst et al., 2024), as well as Grok-3 (xAI, 2025), was evaluated on test sets such as Cora, Citeseer and PubMed. Additionally, open-source LLMs, including Llama 3.3 (Ai, 2024) and Qwen 2.5 (Yang et al., 2024), were fine-tuned and tested in node classification and link prediction tasks to assess their effectiveness. These experiments facilitated a comparative analysis of different LLM architectures in graph-related tasks. Section 5.4 further explores the impact of LLM-based enhancement strategies on model performance across various graph datasets. We also discuss recent graph-LLM and graph-MLLM studies, including Graph-MLLM and MLaGA. Graph-MLLM is primarily a benchmark for multimodal graph learning and evaluates different MLLM usage paradigms. MLaGA targets multimodal graphs with both textual and visual node attributes through a structure-aware multimodal encoder and multimodal instruction tuning. Since most datasets in our evaluation are graph-text or feature-attributed graphs without native visual node attributes, these methods are not directly comparable under all settings. We therefore include them in the related-work and baseline-positioning discussion, and we report direct numerical comparisons only when the required input modalities and task settings match.

The training and evaluation setup: In all evaluations, the model temperature was set to zero. During the training phase, the batch size for all benchmark models was set to 128, and the AdamW (Yao et al., 2021) optimizer was employed (with a learning rate of 0.0002 for the text decoder and 0.00002 for the structural-to-text items). The fine-tuning experiments were conducted on a system configured with a Xeon(R) Platinum 8358P CPU and four 4090 GPUs. For datasets without official splits, we use a 7:3 train and test split and apply the same split to all compared methods. The discrepancies between the model predictions and the actual charts were quantified through exact matching. In subsequent experiments, we employed the LoRA (Hu et al., 2022) method to fine-tune the LLMs, thereby enhancing their performance and that of the structural-to-text items. These were combined with the textual descriptions to be passed to the text decoder, which generated the final answers.

Statistical testing: For each trainable method, we report the mean and standard deviation over multiple independent runs with different random seeds. To support claims of improvement, we consistently use a two-sided Wilcoxon signed-rank test on paired per-run results between M2GNN and the strongest reimplemented baseline under the same data splits and run seeds. We use the Wilcoxon signed-rank test because the number of repeated runs is limited and we do not assume normality of the paired performance differences. Results are considered statistically significant when *p* < 0.05. For baselines whose paired per-run results are unavailable, we report descriptive comparisons only and do not assign statistical significance. No paired t-tests are used for the significance markers reported in the main tables.

LLM inference and API configuration: All closed-source API calls are performed with deterministic inference settings: *temperature* = 0, *top*_*p*_ = 1.0, *frequency*_*penalty*_ = 0, *presence*_*penalty*_ = 0, and *n* = 1. The maximum output length is set to 256 tokens for semantic rewriting and 512 tokens for zero-shot prediction prompts. The maximum input length is limited to 512 tokens; when the node text exceeds this limit, we retain the title, keywords, and the first 384 tokens of the abstract. API-based LLMs are used only for frozen semantic encoding or zero-shot backbone analysis, and their outputs are cached before GNN training. For open-source LLM backbones, we use Llama 3.3 and Qwen 2.5 with bf16 precision and a maximum context length of 1024. LoRA fine-tuning is applied only to the attention projection layers with rank *r* = 16, scaling factor α = 32, and *dropout* = 0.05. The base LLM parameters are frozen unless otherwise stated. For datasets without official splits, the train and test split is generated once with seed 42 and reused by all compared methods. Repeated runs are then performed with multiple independent random seeds for candidate sampling and model initialization. Statistical tests are paired by using the same run seeds for M2GNN and the corresponding reimplemented baseline.

Prompt design and LLM input format: To make the LLM-related implementation reproducible, we use fixed prompt templates for all LLM-assisted components. The prompts are designed to provide task information, node textual attributes, and restricted local structural context, while preventing label leakage from validation and test nodes. Table 4 summarizes the prompt templates used for node semantic encoding, zero-shot LLM node classification, and zero-shot LLM link prediction. The node semantic encoding prompt is used to obtain cached LLM-derived representations for M2GNN, whereas the zero-shot classification and link prediction prompts are used only for LLM-backbone evaluation.

**Table 4:** Prompt templates used in M2GNN.

Purpose	System prompt	User prompt
Node semantic encoding	You are a graph learning assistant. Convert node information into a concise, label-free semantic representation. Do not infer or output the node label.	Dataset: {dataset}. Task: {task}. Node ID: {id}. Node attributes: {text}. Local context: degree={degree}; sampled neighbor keywords={keywords}. Output a compact semantic representation for embedding.
Zero-shot LLM node classification baseline	You are a node classification assistant. Predict the most likely class from the candidate label set based only on the provided node text and local graph context.	Candidate labels: {label_set}. Node attributes: {text}. Local context: {context}. Return exactly one label from the candidate label set.
Zero-shot LLM link prediction baseline	You are a link prediction assistant. Estimate whether two nodes are likely to be connected based on their text and local graph context.	Node A: {text_A}. Node B: {text_B}. Local context: {context_A, context_B}. Return a JSON object: {“link”: 0 or 1, “confidence”: value between 0 and 1}.

Runtime and memory measurement: Runtime and peak GPU memory are measured on the same hardware platform used for the main experiments, consisting of a Xeon(R) Platinum 8358P CPU and NVIDIA RTX 4090 GPUs. For each large-scale dataset, we report the one-time offline LLM cache time, graph refinement or SPE preprocessing time, average training time per epoch, total training time, inference time, and peak GPU memory. GPU memory is measured using the maximum allocated CUDA memory during training. To avoid mixing offline and online costs, cached LLM embeddings and candidate edge scores are generated once and reused in subsequent training and evaluation.

Fairness protocol for LLM-based baselines: To address the comparability of LLM-based baselines, we distinguish four types of comparisons in our experiments. First, Tables 5, 6 report standard graph-learning comparisons against representative GNN, graph structure learning, and graph–language baselines under their commonly reported or reimplemented settings. These results are intended to evaluate the overall effectiveness of M2GNN in the graph-learning setting, but they should not be interpreted as a strictly parameter-matched LLM comparison. Second, Table 7 reports a backbone-controlled comparison, where all LLM-based variants use the same Qwen 2.5-7B LLM encoder, the same prompt template, the same train and test split, the same decoding settings, and the same cached semantic embeddings. This setting directly evaluates the contribution of the proposed semantic edge refinement, stable positional encoding, and adaptive fusion modules under a fixed language backbone. Third, Table 8 reports LLM-backbone sensitivity by varying the language backbone under the same M2GNN framework. Fourth, Table 9 reports LLM parameter-size sensitivity within the Qwen 2.5 model series. Closed-source LLMs such as ChatGPT-4.5, ChatGPT-4o, and Grok-3 are therefore treated as strong semantic encoders or upper-bound API backbones rather than parameter-matched baselines against open-source LLMs.

**Table 5:** ROC AUC for Link Prediction (LP) and F1 score for Node Classification (NC) tasks.

Category	Method	Cora		Citeseer		PubMed		PPI	
		LP	NC	LP	NC	LP	NC	LP	NC
Traditional GNN	GCN (Jiang et al., 2019)	90.4 ± 0.2	81.3 ± 0.3	75.1 ± 0.6	67.9 ± 0.5	91.1 ± 0.5	78.1 ± 0.2	53.2 ± 0.5	51.5 ± 0.6
	GraphSAGE (Hamilton et al., 2017)	85.5 ± 0.6	77.9 ± 2.4	89.1 ± 0.7	75.1 ± 0.3	86.2 ± 1.0	77.4 ± 2.2	65.0 ± 0.7	63.7 ± 0.6
	GAT (Veličković et al., 2018)	93.7 ± 0.1	83.0 ± 0.7	69.5 ± 0.5	72.5 ± 0.7	91.2 ± 0.1	79.0 ± 0.3	90.1 ± 0.7	92.2 ± 0.8
	MLP (Chami et al., 2019)	83.1 ± 0.5	51.5 ± 1.0	66.2 ± 0.5	57.7 ± 0.9	84.1 ± 0.9	72.4 ± 0.2	67.8 ± 0.1	67.1 ± 0.3
Advanced GNN	HGCN (Chami et al., 2019)	92.9 ± 0.1	79.9 ± 0.2	90.1 ± 0.1	78.3 ± 0.1	96.3 ± 0.0	80.3 ± 0.3	83.3 ± 0.5	82.2 ± 0.4
	HyperIMBA (Fu et al., 2023)	89.2 ± 0.4	83.5 ± 0.3	72.2 ± 0.2	75.0 ± 0.4	81.9 ± 0.8	79.1 ± 0.4	81.1 ± 0.4	80.9 ± 0.3
	IDGL (Chen et al., 2020)	88.7 ± 0.5	88.6 ± 0.4	81.2 ± 0.4	80.9 ± 0.1	89.1 ± 0.3	88.3 ± 0.1	83.3 ± 0.2	82.2 ± 0.1
	GAug (Zhao et al., 2021)	87.3 ± 0.6	86.7 ± 0.6	79.0 ± 0.7	77.6 ± 1.0	85.6 ± 0.5	84.5 ± 0.4	81.6 ± 0.6	80.1 ± 0.5
LLM-enhanced	CoGSL (Liu et al., 2022a)	83.9 ± 0.4	82.1 ± 0.5	79.1 ± 0.2	78.8 ± 0.1	85.1 ± 0.3	85.2 ± 0.2	80.1 ± 0.5	79.1 ± 0.9
	WSGNN (Lao et al., 2022)	89.8 ± 0.2	89.6 ± 0.2	82.2 ± 0.5	80.9 ± 0.5	88.5 ± 0.2	87.2 ± 0.3	82.8 ± 0.4	82.1 ± 0.8
	SUBLIME (Liu et al., 2022b)	85.4 ± 0.2	85.0 ± 0.4	47.5 ± 0.4	43.7 ± 7.1	87.2 ± 0.1	86.0 ± 0.3	80.5 ± 0.7	79.1 ± 0.6
	STABLE (Li et al., 2022)	89.5 ± 0.9	88.8 ± 0.4	76.0 ± 1.3	75.7 ± 1.0	87.4 ± 0.6	86.3 ± 0.2	79.2 ± 0.1	77.4 ± 0.3
	Nodeformer (Wu et al., 2022)	89.2 ± 1.4	88.6 ± 1.0	81.6 ± 0.7	80.3 ± 0.6	88.8 ± 0.5	87.9 ± 0.3	84.1 ± 0.3	83.2 ± 0.1
	GSR (Zhao et al., 2023b)	89.2 ± 0.9	87.6 ± 1.2	80.1 ± 1.2	78.8 ± 1.6	86.3 ± 0.7	85.6 ± 0.6	87.2 ± 0.6	86.6 ± 0.5
	SEGSL (Zhou et al., 2023)	88.5 ± 0.4	87.5 ± 0.7	79.2 ± 0.4	78.9 ± 0.5	88.9 ± 0.6	87.6 ± 0.4	82.7 ± 0.5	82.3 ± 0.7
	GraphEdit (Guo et al., 2024)	91.2 ± 0.8	90.9 ± 1.2	82.2 ± 0.8	81.6 ± 1.4	94.2 ± 0.8	94.1 ± 0.3	89.3 ± 0.2	88.8 ± 0.1
	GLEM-GNN (Chen et al., 2024)	89.9 ± 0.5	89.1 ± 0.7	86.2 ± 0.2	85.1 ± 0.4	92.9 ± 0.5	92.6 ± 0.3	88.3 ± 0.3	87.2 ± 0.2
	GLEM-LM (Chen et al., 2024)	83.2 ± 0.4	82.7 ± 1.1	79.2 ± 0.4	78.2 ± 0.6	94.9 ± 0.3	94.4 ± 0.2	86.7 ± 0.8	85.2 ± 0.3
	Ours	94.0 ± 0.2^†^	93.2 ± 0.1^†^	91.2 ± 0.5^†^	87.5 ± 0.5^†^	94.8 ± 0.2	95.7 ± 0.3^†^	91.1 ± 0.3^†^	90.8 ± 0.3

We highlighted the top first, second, and third results per dataset. † Indicates that M2GNN is significantly better than the strongest reimplemented baseline under a two-sided Wilcoxon signed-rank test with *p* < 0.05.

**Table 6:** Performance comparison on large-scale Link Prediction (LP) and Node Classification (NC) datasets.

Method	DBLP(Aminer)		OGBN-ArXiv		Reddit	
	LP	NC	LP	NC	LP	NC
GCN (Wu et al., 2023)	79.4 ± 0.2	72.5 ± 0.4	76.2 ± 0.3	71.7 ± 0.3	93.5 ± 0.1	93.3 ± 0.2
GraphSAGE (Li et al., 2021)	78.5 ± 0.4	77.7 ± 0.2	75.1 ± 0.2	71.5 ± 0.3	94.2 ± 0.2	95.3 ± 0.1
GAT (Wu et al., 2023)	76.2 ± 0.3	72.0 ± 0.4	74.9 ± 0.5	71.9 ± 0.4	90.2 ± 0.1	90.7 ± 0.2
MLP (Wu et al., 2023)	74.1 ± 0.5	72.0 ± 0.5	60.2 ± 0.3	55.5 ± 0.2	87.2 ± 0.2	88.2 ± 0.1
HGCN (Chami et al., 2019)	76.9 ± 0.2	75.1 ± 0.3	69.1 ± 0.4	68.3 ± 0.6	76.2 ± 0.2	77.6 ± 0.3
HAT (Zhang et al., 2021)	75.1 ± 0.5	74.6 ± 0.3	71.2 ± 0.3	70.1 ± 0.1	79.5 ± 0.4	79.2 ± 0.5
H2H-GCN (Dai et al., 2021)	77.0 ± 0.1	75.2 ± 0.4	72.5 ± 0.2	71.4 ± 0.2	79.2 ± 0.6	78.5 ± 0.8
GOAT (Deng et al., 2024)	83.2 ± 0.4	82.0 ± 0.4	72.2 ± 0.5	72.4 ± 0.4	81.2 ± 0.3	80.1 ± 0.3
NAGphormer (Deng et al., 2024)	74.2 ± 0.4	73.6 ± 0.2	72.6 ± 0.5	70.1 ± 0.6	75.1 ± 0.3	72.9 ± 0.4
Nodeformer (Deng et al., 2024)	74.6 ± 0.2	72.9 ± 0.1	69.3 ± 0.5	67.1 ± 0.8	73.3 ± 0.8	72.6 ± 0.6
GSR (Zhao et al., 2023b)	83.2 ± 0.4	81.5 ± 0.2	75.2 ± 0.2	74.7 ± 0.4	82.2 ± 0.1	80.7 ± 0.2
SEGSL (Zhou et al., 2023)	79.2 ± 0.1	77.5 ± 0.3	74.2 ± 0.6	73.8 ± 0.3	78.6 ± 0.7	78.2 ± 0.5
GraphEdit (Guo et al., 2024)	83.2 ± 0.4	82.5 ± 0.2	75.2 ± 0.1	74.0 ± 0.4	82.0 ± 0.1	81.5 ± 0.4
GLEM-GNN (Chen et al., 2024)	84.3 ± 0.2	83.2 ± 0.1	76.5 ± 0.1	75.9 ± 0.2	77.8 ± 0.6	77.0 ± 0.8
GLEM-LM (Chen et al., 2024)	82.6 ± 0.3	81.2 ± 0.2	76.2 ± 0.3	75.7 ± 0.2	77.1 ± 0.4	76.7 ± 0.3
Ours	85.1 ± 0.2^†^	84.3 ± 0.8^†^	77.3 ± 0.5^†^	75.6 ± 0.7	94.4 ± 0.4	94.1 ± 0.2

We highlighted the top first, second, and third results per dataset. † Indicates that M2GNN is significantly better than the strongest reimplemented baseline under a two-sided Wilcoxon signed-rank test with *p* < 0.05.

**Table 7:** Backbone-controlled comparison of LLM-based variants under a fixed Qwen 2.5-7B backbone.

Method	LLM backbone	Trainable LLM?	Cora NC	Citeseer NC	PubMed NC	Cora LP
LLM-only zero-shot	Qwen 2.5-7B	No	52.4 ± 1.5	48.6 ± 1.2	58.3 ± 1.4	55.2 ± 1.6
LLM + MLP	Qwen 2.5-7B	LoRA / No	58.1 ± 1.2	56.4 ± 0.9	64.7 ± 1.1	60.8 ± 1.0
GLEM-LM-style	Qwen 2.5-7B	LoRA	61.5 ± 0.8	60.2 ± 1.0	69.2 ± 0.8	63.4 ± 0.7
GLEM-GNN-style	Qwen 2.5-7B	LoRA	63.8 ± 0.7	62.5 ± 0.8	71.6 ± 0.6	65.1 ± 0.9
M2GNN w/o edge refinement	Qwen 2.5-7B	LoRA	65.0 ± 0.6	64.1 ± 0.7	72.8 ± 0.7	66.8 ± 0.6
M2GNN w/o adaptive fusion	Qwen 2.5-7B	LoRA	65.5 ± 0.5	64.8 ± 0.5	73.1 ± 0.5	67.3 ± 0.8
M2GNN full	Qwen 2.5-7B	LoRA	**67.2** **±** **0.3**	**66.3** **±** **0.6**	**74.5** **±** **0.8**	**69.5** **±** **0.5**

All methods use the same LLM encoder, prompt template, train/test split, decoding settings, and cached semantic embeddings. Bold values indicate the best-performing result for each task.

**Table 8:** Prediction calibration measured by ECE. Lower is better.

Dataset	Task	GraphEdit ECE	GLEM-GNN ECE	Strongest baseline ECE	M2GNN ECE
Cora	NC	0.084	0.076	0.072	0.045
Citeseer	NC	0.092	0.081	0.079	0.052
PubMed	NC	0.075	0.068	0.065	0.038
Cora	LP	0.088	0.082	0.078	0.041
PubMed	LP	0.071	0.065	0.061	0.035
OGBN-Arxiv	NC	0.115	0.102	0.098	0.064
Reddit	NC	0.128	0.114	0.106	0.072

**Table 9:** Impact of LLM parameter size on node classification performance in M2GNN.

	Cora	Citeseer	PubMed
M2GNN with Qwen 2.5-3B	65.0 ± 0.4	63.1 ± 0.1	70.0 ± 0.3
M2GNN with Qwen 2.5-7B	67.2 ± 0.3	66.3 ± 0.6	74.5 ± 0.8
M2GNN with Qwen 2.5-14B	76.3 ± 0.1	75.5 ± 0.8	76.2 ± 0.2
M2GNN with Qwen 2.5-32B	80.1 ± 0.4	79.4 ± 0.3	82.7 ± 0.6
M2GNN with Qwen 2.5-72B	**89.3** **±** **0.5**	**85.2** **±** **0.4**	**91.6** **±** **0.6**

Bold values indicate the best-performing result for each task.

### 5.2 Overall performance

Tables 5, 6 report ROC-AUC for link prediction and F1 score for node classification on standard and large-scale datasets. On Cora and Citeseer, M2GNN achieves the best mean performance for both LP and NC. On PubMed, M2GNN obtains the best NC result and remains competitive on LP. On PPI, M2GNN performs best for LP, whereas GAT achieves a higher NC score, suggesting that the benefit of LLM-derived semantics is less pronounced when natural-language node attributes are limited.

On the large-scale datasets, M2GNN remains best or competitive under cached LLM preprocessing and sparse candidate refinement. The results on DBLP(Aminer), OGBN-Arxiv, and Reddit indicate that the proposed graph-text refinement and fusion strategy can scale to larger static attributed graphs, although the gains are not uniform across all datasets and tasks. Therefore, we interpret M2GNN as a competitive graph-text prediction framework rather than as universally superior to all baselines.

Comparisons involving different LLM backbones are intentionally excluded from the backbone-controlled setting in Table 7. They are analyzed separately in the LLM-backbone sensitivity analysis in Section 5.4 and Table 8, while parameter-size effects within the Qwen 2.5 series are analyzed in Section 5.5 and Table 9. Thus, Tables 5, 6 should be interpreted as overall graph-learning comparisons, not as strictly parameter-matched LLM comparisons.

Overall, Tables 5, 6 show that M2GNN achieves best or competitive performance across the evaluated static graph benchmarks. The two-sided Wilcoxon signed-rank tests show that several observed improvements of M2GNN over the strongest reimplemented baselines are significant at *p* < 0.05. For dataset-task pairs where the Wilcoxon test does not indicate statistical significance, we describe the result as competitive rather than as a definitive improvement. We therefore avoid claiming general superiority over all graph learning settings. In particular, the current experiments do not evaluate billion-scale knowledge graphs or dynamic temporal graphs, which require relation-aware or time-aware modeling beyond the present formulation.

### 5.3 Calibration analysis

In addition to F1 and ROC-AUC, we evaluate prediction calibration using Expected Calibration Error (ECE) (Table 8). For node classification, confidence is defined as the maximum softmax probability. For link prediction, confidence is defined as the predicted probability of the positive edge class. Following common practice, we divide predictions into M confidence bins and compute (Equation 31):


$$\begin{array}{c} \text{ECE}=\overset{M}{\underset{m=1}{\sum }}\frac{\left|B_{m}\right|}{n}|\text{acc}\left(B_{m}\right)-\text{conf}\left(B_{m}\right)|, \end{array}$$ (31)

where *B*_*m*_ is the set of samples whose confidence falls into the *m*-th bin, acc(*B*_*m*_) is the empirical accuracy in that bin, and conf(*B*_*m*_) is the average confidence. We use *M* = 15 bins. Lower ECE indicates better calibration.

The calibration results provide a complementary view to F1 and ROC-AUC. In settings where M2GNN achieves higher accuracy but similar or worse ECE, we avoid claiming that the model is uniformly better. Conversely, when M2GNN improves both predictive performance and ECE, the results suggest that the semantic and structural fusion not only improves ranking and classification accuracy but also yields better calibrated confidence estimates.

### 5.4 Analysis of different LLMs

We analyze how the choice of language backbone affects the performance of M2GNN on node classification. Table 10 compares closed-source LLMs, open-source LLMs, and smaller bidirectional language encoders under the same M2GNN framework. ChatGPT-4.5 achieves the highest mean scores on the evaluated datasets, while ChatGPT-4o obtains similar results on several datasets. Grok-3, Llama 3.3, and Qwen-2.5 also provide competitive semantic representations, but their performance is generally lower than that of ChatGPT-4.5 in this setting.

**Table 10:** Performance comparison of M2GNN with different LLMs in node classification.

	Cora	Citeseer	PubMed	DBLP	OGBN-Arxiv
M2GNN with ChatGPT 4.5	**93.2** **±** **0.1**	**87.5** **±** **0.5**	**95.7** **±** **0.3**	**84.3** **±** **0.8**	**75.6** **±** **0.3**
M2GNN with ChatGPT 4o	92.2 ± 0.2	86.6 ± 0.6	95.1 ± 0.3	83.8 ± 0.4	75.3 ± 0.7
M2GNN with Grok 3	91.8 ± 0.5	85.3 ± 0.2	93.5 ± 0.8	81.2 ± 0.6	74.7 ± 0.1
M2GNN with Llama 3.3	90.2 ± 0.7	82.5 ± 0.8	91.5 ± 0.2	81.5 ± 0.4	71.2 ± 0.6
M2GNN with Qwen 2	89.3 ± 0.5	85.2 ± 0.4	91.6 ± 0.6	80.2 ± 0.2	73.5 ± 0.7
M2GNN with BERT	67.1 ± 0.4	64.5 ± 0.9	72.5 ± 0.4	61.2 ± 0.7	59.2 ± 0.6
M2GNN with ELECTRA	69.0 ± 0.5	68.1 ± 0.2	73.9 ± 0.8	63.2 ± 0.4	60.2 ± 0.1

Bold values indicate the best-performing result for each task.

In contrast, BERT (Jawahar et al., 2019) and ELECTRA (Clark et al., 2020) lead to substantially lower scores across the evaluated datasets. For example, on Cora, BERT and ELECTRA obtain 67.1±0.4 and 69.0±0.5, respectively, whereas M2GNN with ChatGPT-4.5 obtains 93.2±0.1. These results suggest that stronger LLM backbones can provide more useful semantic representations for M2GNN. However, this experiment should be interpreted as a backbone sensitivity analysis rather than a parameter-matched comparison, because closed-source and open-source models differ in parameter scale, pretraining data, and inference settings.

In summary, these experiments highlight the substantial influence of the selected LLM on the performance of M2GNN. These results suggest that the choice of LLM backbone affects M2GNN performance. Larger or stronger LLMs generally provide better semantic representations in our experiments, but the comparison should be interpreted cautiously because closed-source and open-source LLMs differ in parameter scale, training data, and inference settings.

### 5.5 Analysis of parameter size

We conducted experiments using the Qwen 2.5 model series with varying parameter sizes to analyse how the parameter size of an LLM impacts the performance of M2GNN. A clear and consistent trend is observed across all datasets: increasing the parameter size led to substantial performance improvements of M2GNN (Table 9). Specifically, on the Cora dataset, the accuracy significantly increased from 65.0 ± 0.4 with the smallest model (3B parameters) to 89.3 ± 0.5 with the largest model (72B parameters). Similar improvements were observed on the Citeseer and PubMed datasets, with accuracy rising from 63.1 ± 0.1 and 70.0 ± 0.3 respectively (3B parameters), to 85.2 ± 0.4 and 91.6 ± 0.6, respectively (72B parameters). This improvement trend emphasizes that larger LLMs provide richer semantic representations, which benefits the node classification task within our M2GNN framework. These results underline the importance of using powerful LLMs to achieve optimal performance in graph-related tasks.

### 5.6 Analysis of LLM contribution and fusion strategies

**Figure 3:**
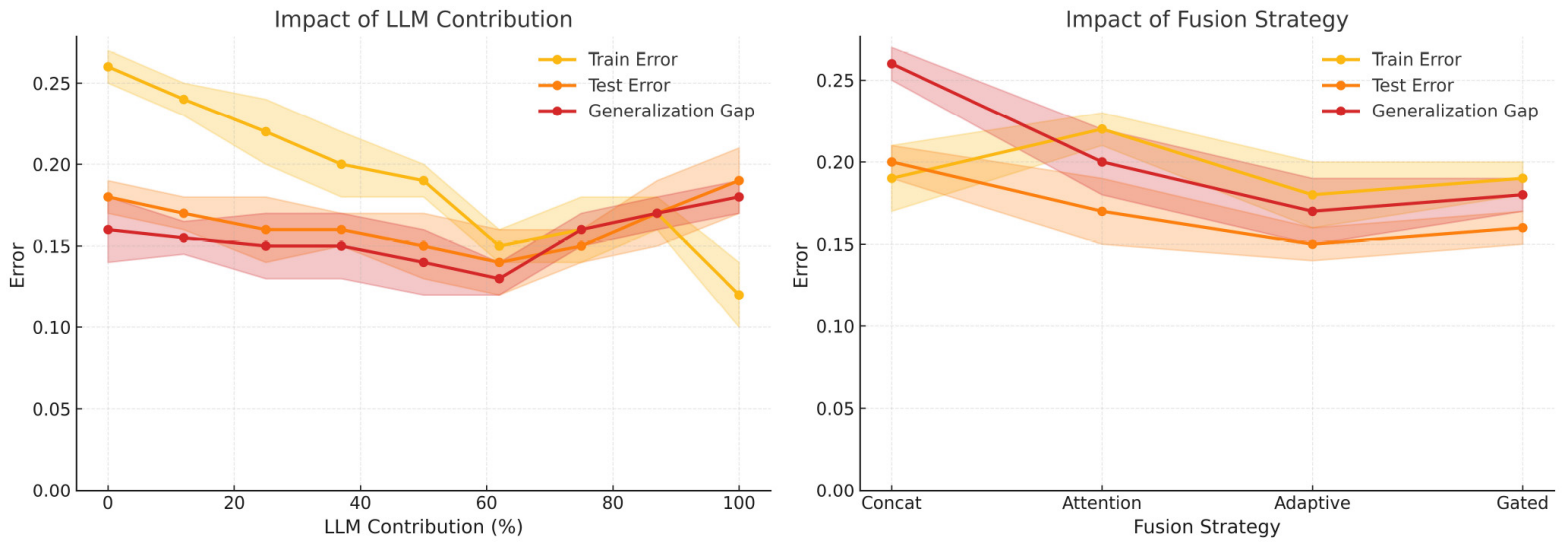
Impact of LLM contribution and fusion strategies on model performance. Training error, test error, and generalization gap vs. LLM contribution and fusion strategy.

We conducted additional experiments to analyse the effect of varying fusion proportions between LLM and GNN contributions as well as different multimodal data fusion strategies on M2GNN's performance. Figure 3 presents the results. Firstly, examining the impact of the fusion ratio between LLM and GNN revealed that increasing the contribution of LLM reduced the training and test errors up to a certain threshold. Notably, Intersections between the training and test error curves around the 60% contribution point underscore the significance of achieving an optimal balance between structural (GNN) and semantic (LLM) information. This overlap highlights a region where the model generalizes most effectively, suggesting that neither purely structural nor purely semantic data alone can optimally address multimodal tasks. Secondly, evaluating the multimodal fusion strategies (Concat, Attention, Adaptive, and Gated), we observed that the Adaptive fusion strategy achieved the lowest training error, test error, and generalization gap compared to other strategies. The Concat strategy, although simpler, exhibited higher errors and a larger generalization gap, suggesting less effective integration of multimodal information. The Attention and Gated fusion strategies delivered intermediate performances, with Adaptive slightly outperforming Attention.

These findings demonstrate that an appropriate balance between LLM and GNN contributions, as well as an effective multimodal fusion strategy, critically influences the overall performance and generalization capabilities of the M2GNN model. Based on these experimental results, we selected the optimal fusion ratio (approximately 60% LLM contribution) and the Adaptive fusion strategy for our final model implementation, ensuring superior performance and effective integration of multimodal information.

### 5.7 Ablation study

We conducted two separate ablation studies on the Cora dataset to comprehensively evaluate the significance of different modules and data types within our M2GNN framework. Firstly, we assessed the individual contributions of the GNN and LLM modules. The absence of the GNN module resulted in a 12.19% decrease in performance, while removing the LLM module led to a 4.46% performance drop (Table 11). This illustrates that both modules play crucial roles in the model, with GNN having slightly more impact on the overall performance. Secondly, we analyzed the importance of text and graph data individually. Excluding graph structural data led to a substantial decrease of 14.36% in model performance, whereas omitting textual data resulted in a decrease of 5.76% (Table 12). This finding highlights the indispensable role of structural graph information, alongside textual content, in achieving high accuracy in node classification and link prediction tasks. These ablation studies underscore the need to integrate LLM and GNN modules and utilize multimodal data (textual and structural) to fully exploit the potential of the M2GNN framework.

**Table 11:** Branch-level ablation of the LLM and GNN predictors used in prediction-level fusion (Equation 29) on the Cora dataset for node classification.

Method	Cora	Δ_*M*2*GNN*_
w/o GNN	0.8103 ± 0.0013	↓ 12.19%
w/o LLM	0.8877 ± 0.0021	↓ 4.46%
M2GNN	0.9322 ± 0.0022	–

**Table 12:** Modality-level ablation of graph and text information used in feature-level fusion (Equation 22) on the Cora dataset for link prediction.

Method	Cora	Δ_*M*2*GNN*_
w/o Graph	0.7965 ± 0.0012	↓ 14.36%
w/o Text	0.8825 ± 0.0038	↓ 5.76%
M2GNN	0.9401 ± 0.0023	–

The ablation results further clarify the separate roles of the two fusion stages. Table 12 provides modality-level evidence for the feature-level fusion stage in Equation 22. Removing graph structural information or textual information weakens performance, indicating that the fused representation $h_{i}^{fused}$ benefits from both structural and semantic inputs. Table 11 provides branch-level evidence for the prediction-level fusion stage in Equation 29. Removing either the GNN predictor or the LLM predictor degrades performance, indicating that the final gate benefits from combining complementary structural and semantic predictors rather than relying on a single branch. Therefore, Equation 22 contributes mainly to shared graph-text representation learning, whereas Equation 29 contributes mainly to final prediction calibration by adaptively weighting the two predictor outputs.

### 5.8 Effect of depth on performance and over-smoothing

**Figure 4:**
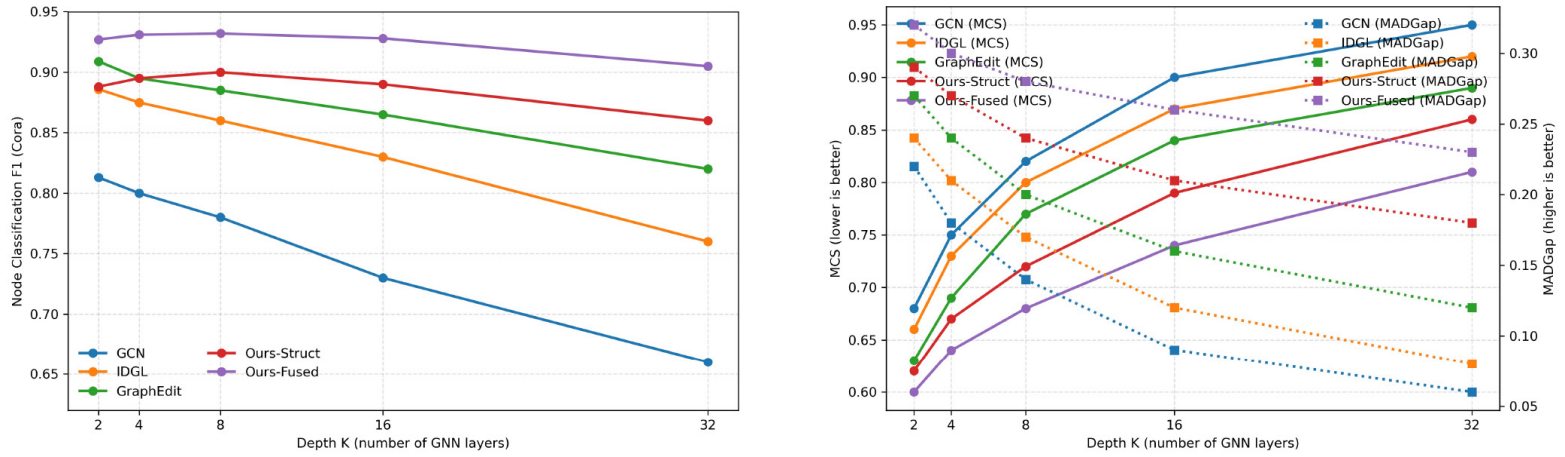
**(a)** Test performance vs. depth *K* on three benchmark datasets for Cora dataset. **(b)** Over-smoothing indicators vs. depth *K* for Cora dataset.

Following Rusch et al. (2023), we treat oversmoothing as the progressive convergence of node representations as model depth increases, and we measure it through representation similarity rather than claiming a complete spectral proof. Specifically, we report mean cosine similarity and MADGap as empirical indicators of whether node embeddings collapse into less distinguishable representations. To assess the robustness of M2GNN under increasing depth, we evaluated all methods with *K* ∈ {2, 4, 8, 16, 32} layers on the Cora dataset. Figure 4a shows node classification *F1* scores, and Figure 4b reports over-smoothing indicators: mean cosine similarity (MCS, lower is better) and MADGap (higher is better). For M2GNN, we compare the *Ours-Struct* variant, using only the structural encoder, and the full *Ours-Fused* model with adaptive multimodal fusion.

GCN peaks at shallow depths (*K* = 2–4) but degrades rapidly, reflecting over-smoothing. IDGL and GraphEdit slow this decline, with GraphEdit retaining better deep-layer accuracy. *Ours-Struct* surpasses all baselines beyond *K* = 8, while *Ours-Fused* achieves the highest scores overall, sustaining competitive accuracy even at *K* = 32.

As shown in Figure 4b, MCS rises and MADGap falls sharply for baselines at large *K*, indicating embedding collapse. Both M2GNN variants empirically mitigate this trend, with Ours-Fused yielding lower MCS and higher MADGap at larger depths. These results indicate that the semantic branch and adaptive fusion help preserve representation separation, but they should be interpreted as empirical evidence of oversmoothing mitigation rather than a formal proof that oversmoothing is eliminated.

### 5.9 Visual analysis

**Figure 5:**
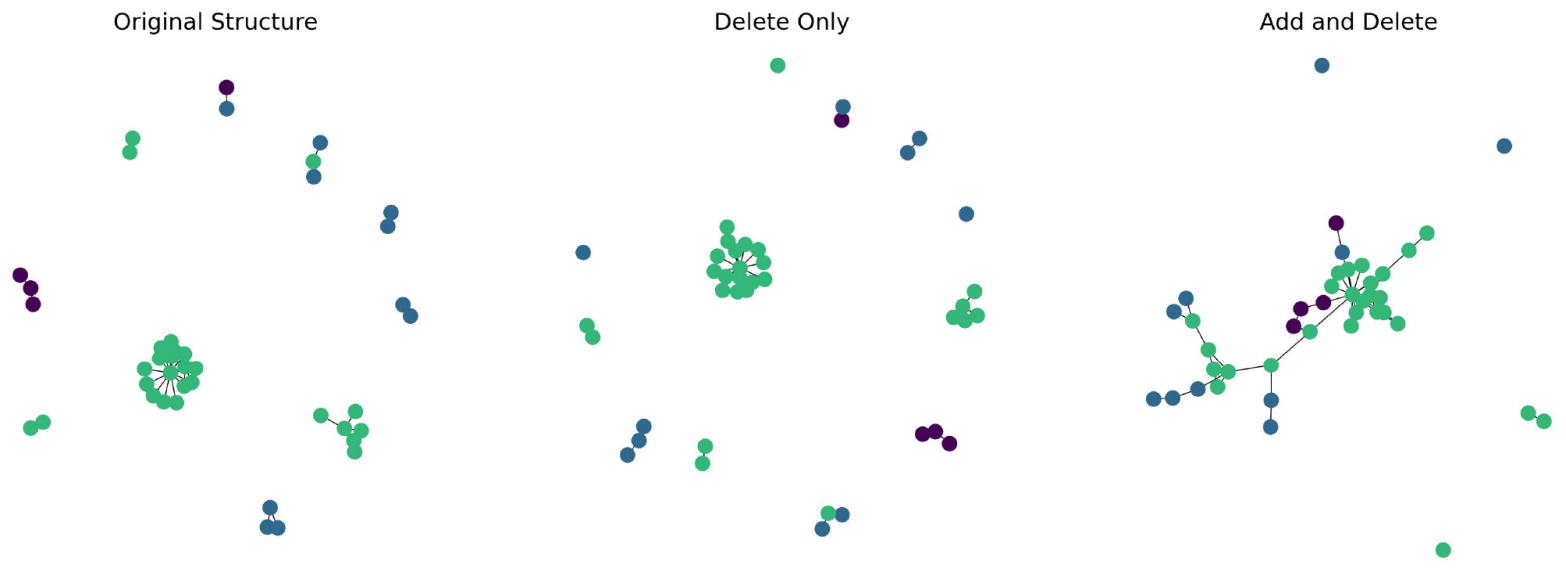
Structural modifications in graph data: impact of edge deletion and addition. Visual analysis of randomly sampled 20 nodes and their one-hot encoded neighbors in the PubMed dataset.

We conducted a visualization analysis to interpret and analyse the structural modifications performed by our graph editing module (Figure 5). By randomly sampling 20 nodes from the PubMed dataset, we visualized their one-hot encoded neighbors under different structural modification strategies: original structure, edge deletion only, and combined edge addition and deletion. In the original structure, nodes displayed scattered distributions, highlighting a typical sparsity and isolation pattern within the graph. When only edge deletion was applied, clusters became more compact and increasingly isolated, indicating potential risks of disconnected subgraphs. Conversely, the combined edge addition and deletion strategy generated more interconnected structures, significantly enhancing local connectivity and cohesion. This visual comparison demonstrates that our graph editing module effectively balances local graph structure modifications, optimizing connectivity and robustness for improved M2GNN performance.

### 5.10 Case study

We conducted a case study to demonstrate the practical effectiveness of our M2GNN framework on the PubMed dataset (Table 13). Given two research papers (Node 2601 and Node 6289), M2GNN was tasked to predict if they belong to the same category among Diabetes Mellitus Type 1, Type 2, or Experimental. The ground truth indicates both nodes represent Type 1 Diabetes papers. Our M2GNN model predicted the correct category with high confidence (89.7%), demonstrating its ability to leverage textual and structural information for accurate and confident predictions in real-world scenarios.

**Table 13:** Case study of M2GNN on PubMed data.

Query: Given two research papers (Node 2601 and Node 6289), the goal is to predict whether they belong to the same category among Diabetes Mellitus Type 1, Type 2, or Experimental.
GT: True (Both are Type 1 Diabetes Papers)
The prediction results of our M2GNN: True (Both are Type 1) Confidence: 89.7%

### 5.11 Ethical considerations and limitations

Ethical considerations and limitations. M2GNN uses LLM-derived semantic information to refine graph structure and support prediction. This design may introduce or amplify biases from two sources. First, LLM embeddings may reflect biases present in pre-training corpora, which can affect semantic similarity estimation and downstream predictions. Second, graph refinement may amplify existing structural inequalities in social, recommendation, or citation networks if the model preferentially adds edges among already well-represented or high-degree nodes. Such effects could reinforce homophily, reduce exposure for minority groups, or increase unfairness in sensitive applications.

To reduce these risks, we use deterministic LLM inference with temperature set to zero, restrict graph edits through confidence thresholds and per-node edit budgets, and keep the accepted edge edits auditable. We also avoid using protected demographic attributes in the evaluated benchmarks. Nevertheless, these mechanisms do not eliminate bias. Deploying M2GNN in high-stakes domains such as healthcare, employment, finance, or social recommendation would require task-specific fairness evaluation, bias auditing, human oversight, and calibration analysis. We therefore present M2GNN as a research framework for graph–text learning rather than as a ready-to-deploy decision system for sensitive real-world settings.

## 6 Conclusion

In this paper, we proposed M2GNN, a graph-text learning framework that integrates LLM-derived semantic information with GNN-based structural representations for node classification and link prediction. M2GNN combines semantic edge refinement, stable positional encoding, feature-level graph-text fusion, and prediction-level gating within a unified prediction pipeline. Experimental results show that M2GNN achieves competitive or improved performance in several evaluated settings, with statistically significant gains under paired two-sided Wilcoxon signed-rank tests in matched comparisons where paired per-run results are available. The backbone-controlled comparison and sensitivity analyses further indicate that the proposed graph-text refinement and fusion modules contribute beyond the choice of a strong LLM encoder.

Despite these results, the current study focuses on static attributed graphs and should not be interpreted as a general solution for billion-scale knowledge graph completion, dynamic temporal graph modeling, or native visual-textual graph reasoning. Extending M2GNN to relation-aware knowledge graphs, temporal graphs, and native visual-textual graph benchmarks will require different modeling assumptions and task-specific objectives. Future work will investigate parameter-efficient adaptation, fairness auditing, calibration, explainability, and broader evaluation under dynamic and multimodal graph settings.

## Data Availability

The original contributions presented in the study are included in the article/supplementary material, further inquiries can be directed to the corresponding author.
